# Depsidomycins B and C: New Cyclic Peptides from a Ginseng Farm Soil-Derived Actinomycete

**DOI:** 10.3390/molecules23061266

**Published:** 2018-05-25

**Authors:** Yun Kwon, Woong Sub Byun, Byung-Yong Kim, Myoung Chong Song, Munhyung Bae, Yeo Joon Yoon, Jongheon Shin, Sang Kook Lee, Dong-Chan Oh

**Affiliations:** 1 Natural Products Research Institute, College of Pharmacy, Seoul National University, Seoul 08826, Republic of Korea; kisi2016@snu.ac.kr (Y.K.); sky_magic@naver.com (W.S.B.); baemoon89@snu.ac.kr (M.B.); shinj@snu.ac.kr (J.S.); sklee61@snu.ac.kr (S.K.L.); 2 ChunLab, Inc., JW TOWER, Seocho-gu, Seoul 06725, Republic of Korea; greg6044@gmail.com; 3 Department of Chemistry and Nanoscience, Ewha Womans University, Seoul 03760, Republic of Korea; smch517@ewha.ac.kr (M.C.S.); joonyoon@ewha.ac.kr (Y.J.Y.)

**Keywords:** farm soil, actinomycete, *Streptomyces* sp., cyclic peptide, antimetastatic

## Abstract

LC/MS-based chemical profiling of a ginseng farm soil-derived actinomycete strain, *Streptomyces* sp. BYK1371, enabled the discovery of two new cyclic heptapeptides, depsidomycins B and C (**1** and **2**), each containing two piperazic acid units and a formyl group at their *N*-terminus. The structures of **1** and **2** were elucidated by a combination of spectroscopic and chemical analyses. These new compounds were determined to possess d-leucine, d-threonine, d-valine, and *S*-piperazic acid based on the advanced Marfey’s method and a GITC (2,3,4,6-tetra-*O*-acetyl-β-d-glucopyranosyl isothiocyanate) derivatization of their hydrolysates, followed by LC/MS analysis. Depsidomycins B and C displayed significant antimetastatic activities against metastatic breast cancer cells (MDA-MB-231).

## 1. Introduction

**Figure 1:**
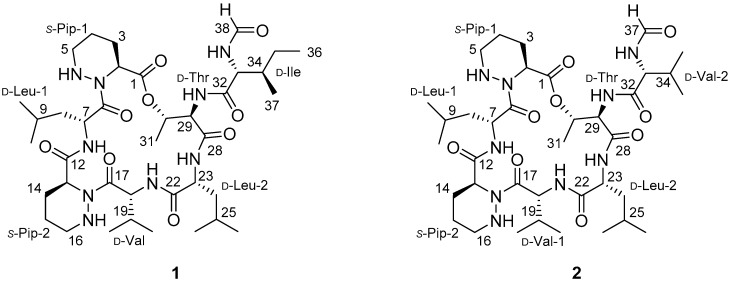
The chemical structures of depsidomycins B and C (**1** and **2**).

Secondary metabolites from soil bacteria have been prolific sources of bioactive natural products for the last century, and they have provided a number of important clinical drugs [1]. However, exhaustive studies of soil bacteria with conventional strategies involving bioassay-guided fractionation have shown decreasing success rates in the discovery of new bacterial secondary metabolites. The recent development of chemical analysis-based search methods has enabled the efficient discovery of new bioactive compounds from bacteria [2]. Our time-course chemical analysis of bacterial metabolites using LC/MS chemical profiling yielded new chemotypes, demonstrating the value of chemical analysis-based research in natural products chemistry. A recent example of the success of such a method is the discovery of a novel pyrazolone-bearing metabolite from a *Streptomyces* strain collected in an intertidal mudflat [3]. A *Deinococcus* strain isolated from the gut of the carpenter ant, *Camponotus japonicus*, was found to produce new aminoglycolipids [4]. New naphthoquinone-oxindoles with a novel carbon framework were discovered from a gut bacterial strain of the dung beetle, *Copris tripartitus* [5]. For the efficient discovery of new bioactive small molecules from bacteria, a new paradigm of search strategies could be used to investigate poorly studied bacterial habitats. Farm soil appears readily accessible but has been essentially abandoned in natural product discovery, possibly because the environment can be regarded as anthropogenic rather than natural. However, the rhizosphere, a narrow region of farm soil, is a unique environment harboring microbial communities that interact with plant root exudates, which could allow these strains to produce unique secondary metabolites [6]. One of our pioneering studies on farm soil bacterial metabolites based on detailed LC/MS chemical profiling resulted in the discovery of a rare norditerpenoid with antibacterial activity from *Actinomadura* sp. inhabiting an experimental farm in the United Kingdom [7]. Our ongoing search for bioactive compounds from farm soil bacteria led us to investigate ginseng farm soil-derived actinomycetes. Ginseng, from the genus *Panax*, is considered one of the most valuable medicinal plants in many Asian countries including Korea [8]. Even though the chemistry of ginseng has been exhaustively studied because of its bioactive compounds [9], the chemistry of the bacterial communities of ginseng farm soil have not been examined. We collected soil from a farm cultivating *Panax ginseng* in Cheorwon, Gangwon-do, Republic of Korea. More than 100 bacterial strains were isolated from the ginseng farm soil. Chemical analyses of the bacteria were performed with a focus on the actinobacterial strains, which are the most prolific bacterial group for the production of small molecules [1]. Time-course chemical analyses of the actinobacterial strains conducted by LC/MS detected multiple unique metabolites with molecular ions ([M + H]^+^) at *m*/*z* 792 and 778 from *Streptomyces* sp. BYK1371. Further large-scale culture and subsequent chemical analysis enabled the elucidation of the structures of these new compounds, and the compounds were named depsidomycins B and C (**1** and **2**, Figure 1). Here, we report their structure determination and biological activity.

## 2. Results and Discussion

### 2.1. Structure Elucidation

Depsidomycin B (**1**) was isolated as a white, amorphous powder. The molecular formula of **1** was found to be C_38_H_65_N_9_O_9_ on the basis of its HR-ESI-MS data. The ^13^C NMR data confirmed the presence of 38 carbons, namely, eight carbonyl carbons (δ_C_ 161.8–175.9), one oxygen-bearing aliphatic carbon (δ_C_ 71.4), nine nitrogen-bearing carbons (δ_C_ 47.8–56.2), and 20 aliphatic carbons (δ_C_ 41.4–11.9) (Table 1). Comprehensive analysis of the ^1^H, ^13^C, and HSQC NMR spectroscopic data of **1** revealed the presence of seven D_2_O exchangeable protons (δ_H_ 7.62, 7.59, 7.52, 7.49, 7.07, 4.86, and 3.95), one formyl functional group (δ_H_ 8.27; δ_C_ 161.8), nine aliphatic methylenes, twelve aliphatic methines, and nine methyl groups. The five downfield, heteroatom-bound protons (δ_H_ 7.62–7.07) and eight carbonyl carbons in the amide/ester carbon chemical shift range indicated that depsidomycin B could be a modified peptide decorated with a formyl functional group. Eight of the eleven double bond equivalents inherent in the molecular formula of **1** could be attributed to carbonyl groups, which indicated the existence of three rings in the structure of depsidomycin B (**1**).

After establishing 1-bond ^1^H-^13^C correlations, structural fragments were assembled based on combined analysis of the COSY, TOCSY, and HMBC NMR spectra. An array of COSY and TOCSY correlations from H-2 to 5-NH through H_2_-3, H_2_-4, and H_2_-5 identified a four-carbon spin system building piperazic acid. In addition, one more piperazic acid unit was established from the COSY correlations among H-13, H_2_-14, H_2_-15, H_2_-16, and 16-NH, along with the corresponding HMBC signals. Continuous analysis of the COSY, TOCSY and HMBC NMR spectra constructed the other amino acids present, namely, an isoleucine, a valine, a threonine, and two leucine amino acid residues. Therefore, depsidomycin B (**1**) possesses eight discrete spin systems composed of two piperazic acids (Pip-1 and Pip-2), two leucines (Leu-1 and Leu-2), an isoleucine (Ile), a valine (Val), a threonine (Thr), and a formyl group.

**Figure 2:**
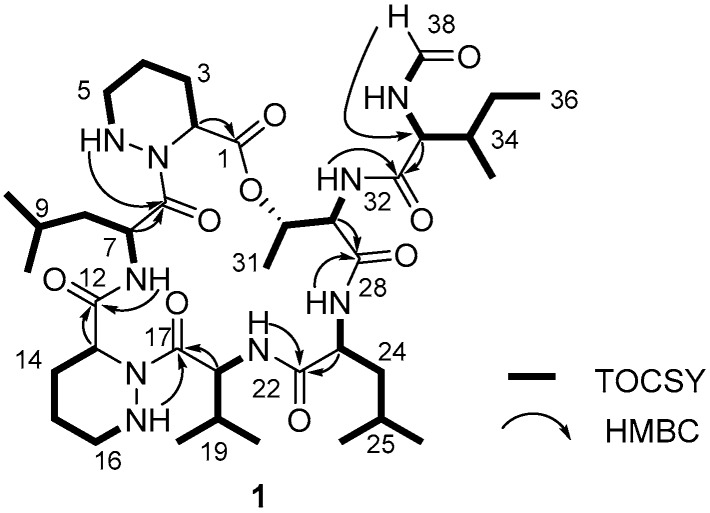
The ^1^H-^1^H TOCSY (bold line) and key HMBC (arrow) correlations of depsidomycin B (**1**).

Based on the eight partial structures and the molecular formula, dereplication of depsidomycin B (**1**) indicated that this compound could have the same planar structure as the previously reported depsidomycin [10,11]. The sequence (Pip-1-Leu-1-Pip-2-Val-Leu-2-Thr-Ile-formamide) of the amino acid residues was confirmed by ^1^H-^13^C HMBC correlations (Figure 2). The formyl functional group was connected to Ile by 33-NH/H-38 COSY and H-38/C-33 HMBC correlations (Figure 2).

**Figure 3:**
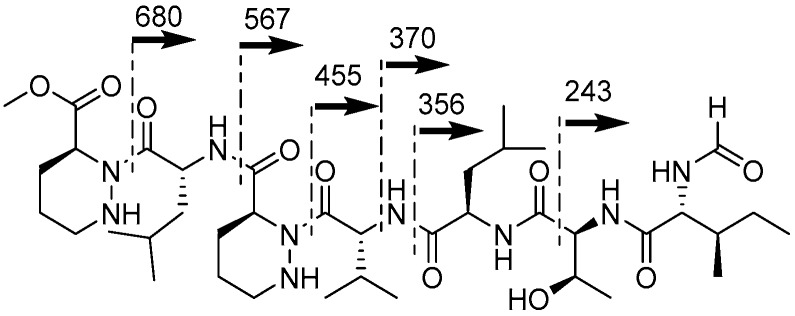
MS/MS fragmentation of the methanolysis product (**3**) of **1**.

The molecular formula (C_38_H_65_N_9_O_9_) of depsidomycin B indicated 11 double bond equivalents. Eight carbonyl carbons and two piperazic acid rings accounted for 10 out of the 11 unsaturations, indicating one additional ring was required in the structure of **1**. The 1D and 2D NMR spectra collected in the aprotic solvents acetone-*d*_6_ and pyridine-*d*_5_ did not show a signal for the hydroxy proton at the β-position of Thr, which implied that a tethered lactone ring could be connected to this unit. In addition, the ROESY NMR cross-peak between H-2 and H-30 (δ_H_ 4.91) supported the existence of a macrocyclic ring connecting Pip-1 and Thr. However, a key HMBC signal from H-30 to C-1 (δ_C_ 169.3), which could unequivocally confirm the presence of such a ring, was not detected in the NMR spectra acquired in these solvents. Therefore, we derivatized depsidomycin B (**1**) by methanolysis and comprehensively analyzed the 1D and 2D NMR spectra of the derivative (**3**) (Figures S22–S27). In the HMBC NMR spectrum of the methanolysis product, the methoxy protons (H-39, δ_H_ 3.74) showed clear correlations with C-1 (δ_C_ 172.1), establishing the connectivity between Pip-1 and Thr in depsidomycin B (**1**) through an ester functional group (Table S2). The sequence of the amino acid residues was also confirmed based on the MS/MS spectrum (fragmentation ions at *m*/*z* 243, 356, 370, 455, 567, and 680) of the methanolysis product (Figure 3 and Figure S28). Overall, the planar structure of depsidomycin B (**1**) was determined to be a cyclic heptapeptide bearing a formamide attached at d-Ile moiety.

The planar structure of **1** was identical to that reported previously for depsidomycin, which showed antibacterial activity [10,11]. A careful comparison of our NMR data of depsidomycin B with the reported data revealed that most of the ^1^H and ^13^C chemical shifts are very similar (within 0.02 and 0.2 ppm, respectively; Table S3). However, the chemical shifts of 7-NH (δ_H_ 7.62), 18-NH (δ_H_ 7.59), 23-NH, and 33-NH (δ_H_ 7.49) in the ^1^H NMR spectra of the two compounds varied by 0.3 ppm. More strikingly, the chemical shifts of 29-NH and H-33 were substantially different; they varied by 0.17 and 0.10 ppm, respectively. The ^13^C NMR chemical shifts at C-13 (δ_C_ 52.8), C-34 (δ_C_ 38.1), and C-36 (δ_C_ 11.8) varied by 0.3, 0.4, and 0.3 ppm, respectively. Moreover, the specific rotation of depsidomycin B (−1.7) was significantly different from that of depsidomycin (−59.7). These spectroscopic differences strongly indicated that depsidomycin B (**1**) is a stereoisomer of depsidomycin, and its amino acid residues are in different configurations; however, fully elucidating these differences requires extensive stereochemical determinations (see Section 2.2).

Depsidomycin C (**2**) was purified as a white powder. Its HR-ESI-MS data confirmed a molecular formula of C_37_H_63_N_9_O_9_, which has one fewer methylene than does depsidomycin B (**1**). The 1D and 2D NMR spectroscopic data of **2** suggested that its NMR spectroscopic features were quite similar to those of **1**, indicating that depsidomycin C (**2**) is structurally analogous to depsidomycin B (**1**). Comprehensive analysis of its 2D NMR spectra revealed that the isoleucine unit of **1** had been replaced with a valine (Val) in **2** (Table 1). Thus, the structure of depsidomycin C (**2**) was elucidated to be a new cyclic heptapeptide with the sequence Pip-1-Leu-Pip-2-Val-1-Leu-2-Thr-Val-2-formamide.

### 2.2. Determination of the Absolute Configurations of Depsidomycins B and C

The absolute configurations of the amino acids were determined by advanced Marfey’s method [12,13] and 2,3,4,6-tetra-*O*-acetyl-β-d-glucopyranosyl isothiocyanate (GITC) derivatization followed by LC/MS analysis [14]. The acid hydrolysate of **1** was derivatized with Marfey’s reagents, *N*-(5-fluoro-2,4-dinitrophenyl)-l-alanine amide (l-FDAA) and d-FDAA. The elution sequences of the l- and d-FDAA derivatives (elution order: d→l for Val, Leu, Thr, and Ile) in the LC/MS chromatography established that the valine, leucine, threonine and isoleucine residues are in the d-configuration (Table S4, Figure S29). Piperazic acid causes the d-FDAA derivatives to elute before the l-FDAA derivatives when it is in the *S*-configuration [15]. In the LC/MS chromatogram, the d-FDAA adduct of the piperazic acid units eluted faster than did the l-FDAA derivative, which confirmed the two piperazic acids in **1** were in the *S*-configuration (Table S4). The absolute configurations of the β-carbons of d-threonine and d-isoleucine were assigned by GITC derivatization and LC/MS analysis. The chromatogram of the GITC adducts of the hydrolysate of depsidomycin B (**1**) displayed retention times consistent with those of d-Thr and d-Ile rather than d-*allo*-Thr and d-*allo*-Ile. This assignment was further confirmed by co-injecting the GITC derivatives of authentic d-Thr, d-*allo*-Thr, d-Ile, and d-*allo*-Ile individually with the GITC derivative of **1**. Based on advanced Marfey’s analysis and GITC derivatization, the absolute configurations of the amino acid units common to **1** and **2** were identical (Figures S30–S32). The additional valine residue in depsidomycin C (**2**), which replaced the d-Ile unit in **1**, was determined to be d-Val.

Overall, depsidomycin B (**1**) was determined to contain d-valine, d-threonine, d-isoleucine, two d-leucines and two *S*-piperazic acids, whereas depsidomycin contains d-valine, l-threonine, l-*allo*-isoleucine, l-leucine, d-leucine, and two configurationally-undetermined piperazic acids. The differences in the NMR chemical shifts (Table S3) and the optical rotations of depsidomycin B and depsidomycin, which have identical planar structures, could be explained by the differences in their stereochemistries.

### 2.3. Biological Activity of Depsidomycins B and C

**Figure 4:**
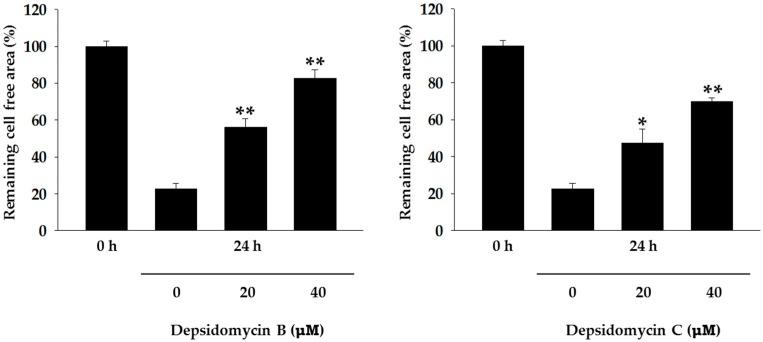
Remaining cell-free area (%) of MDA-MB-231 cells treated with depsidomycin B (**1**) and C (**2**). All data are expressed as the means ± SD (*n* = 3). * *p* < 0.05. ** *p* < 0.01 compared to the control.

**Figure 5:**
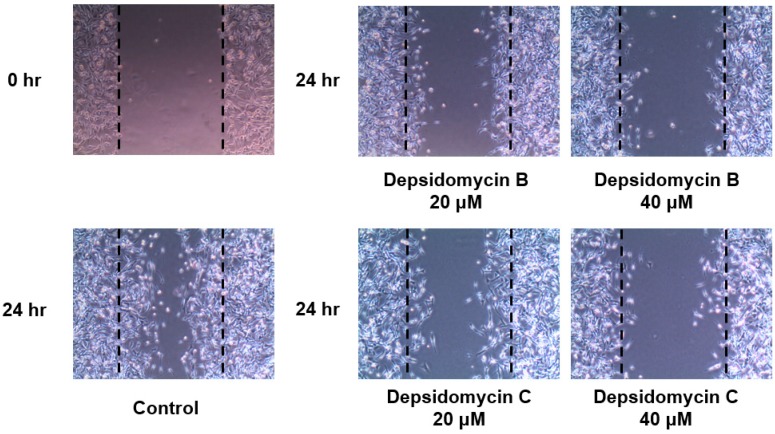
Antimetastatic activity against the breast cancer cell line MDA-MB-231.

Even though depsidomycin was originally reported to show antibacterial activity against *Micrococcus luteus*, depsidomycins B and C did not exhibit antibacterial activity against *M. luteus* in our tests. These cyclic peptides did not inhibit the growth of any in vitro-tested bacteria (Gram positive: *Staphylococcus aureus* ATCC 25923, *Enterococcus faecalis* ATCC 19433, and *Enterococcus faecium* ATCC 19434; Gram negative: *Micrococcus luteus* ATCC 9341, *Klebsiella pneumoniae* ATCC 10031, *Salmonella enterica* ATCC 14028, *Escherichia coli* ATCC 25922) and fungi (*Aspergillus fumigatus* HIC 6094, *Trichophyton rubrum* NBRC 9185, *Trichophyton mentagrophytes* IFM 40996, and *Candida albicans* ATCC 10231) [16], or cancer cell lines (MDA-MB-231, A549, HCT116, SK-HEP-1, SNU638, and K562) [17]. Therefore, their biological activities were further evaluated with respect to their antimetastatic activities against MDA-MB-231 cells (a metastatic breast cancer cell line) in a wound-healing assay [18]. After mechanically generating scratches in MDA-MB-231 cells, the cells were treated with vehicle or depsidomycins B and C for 24 h. The wounds were photographed at 0 h (immediately after wounding) and 24 h with an inverted microscope. Compared to the vehicle-treated group, cell migration (antimetastatic effect) was suppressed in the depsipeptide-treated groups. The remaining cell-free area (%) calculated 24 h after wounding of the vehicle-treated group was 22%, while those of the depsidomycin B-treated groups (20 and 40 µM) were 56% and 82%, respectively (Figure 4 and Figure 5). Depsidomycin C displayed less potent activity, as it provided 47% and 70% remaining cell-free area at 20 and 40 µM, respectively (Figure 4 and Figure 5). This result indicated that depsidomycins B and C (**1** and **2**) have significant dose-dependent antimetastatic effects on the metastatic breast cancer cell line MDA-MB-231 in vitro.

## 3. Experimental Section

### 3.1. General Experimental Procedures

The optical rotations were recorded with a JASCO P-1020 polarimeter. UV spectra were measured on a Perkin-Elmer (Waltham, MA, USA) Lambda 35 UV/VIS spectrometer. IR spectra were recorded on a JASCO FT-IR-4200 spectrometer. ^1^H NMR (850 MHz, 600 MHz) and ^13^C NMR (212.5 MHz, 150 MHz) spectral data were obtained on a Bruker (Billerica, MA, USA) Avance 3 HD and Avance-600 at NCIRF (National Center for Inter-University Research Facilities, Seoul, Republic of Korea). Low-resolution LC/MS data were acquired on an Agilent Technologies (Santa Clara, CA, USA) 1200 series HPLC coupled with an Agilent Technologies (Santa Clara, CA, USA) 6130 quadrupole mass spectrometer. High-resolution electrospray ionization mass spectrometry (HR-ESI-MS) data were collected with a Thermo (Waltham, MA, USA) Finnigan LTQ high-resolution mass spectrometer at NICEM (National Instrumentation Center for Environmental Management, Seoul, Republic of Korea). LC-MS/MS data were obtained with an AB SCIEX Q-TOF 5600 at NICEM and a Waters (Milford, MA, USA) XEVO G2S Q-TOF mass spectrometer at Ewha Womans University (Seoul, Republic of Korea).

### 3.2. Bacterial Isolation

The soil samples were collected from a four-year ginseng farm in Cheorwon, Gangwon-do, Republic of Korea. The samples were dried at room temperature (rt) for 5 h, and the dry soil (1 g) was mixed with 8 mL of sterilized water. The supernatant of the mixture was inoculated onto TWYE agar (TWYE: 1 L of tap water, 18 g of agar, 0.25 g of yeast extract and 0.5 g of K_2_HPO_4_) and then spread. Colonies were repeatedly isolated onto fresh agar plates to obtain single strains. The strain BYK1371 (GenBank accession no. MH037157) was phylogenetically identified as a *Streptomyces* sp. by the phylogenetic tree based on 16S rRNA gene sequence analysis. It is most closely related to *Streptomyces vinaceus* NRBC 13425 and *Streptomyces cirratus* NRRL B-3250 (Figure S35).

### 3.3. Cultivation and Extraction of the Bacterial Strain

The BYK1371 strain was cultured on solid YEME medium (4 g of yeast extract, 10 g of malt extract, 4 g of glucose, and 18 g of agar per 1 L of sterilized water) at 27 °C. The culture was then transferred to 50 mL of liquid YEME medium in a 125 mL Erlenmeyer flask. After incubation at 25 °C with shaking at 180 rpm for 2 days, 20 mL of the culture was inoculated into each of the 2.8 L Fernbach flask containing 1 L of YEME liquid medium. The cultures were cultivated at 27 °C with shaking at 140 rpm for five days because the yields of **1** and **2** were the best for harvest based on the time-course LC/MS analysis of the strain (Figure S34). In total, 85 L of culture was prepared for the isolation of **1** and **2**. The combined total culture (85 L) was extracted twice with 200 L of EtOAc using a 3-L separation funnel. After adding anhydrous sodium sulfate to remove the residual water, the EtOAc layer was concentrated in vacuo to yield 13.2 g of extract.

### 3.4. Isolation of Depsidomycin B *(**1**)* and C *(**2**)*

An aliquot (4.3 g) of the extract was resuspended in MeOH and dried onto Celite in vacuo to generate a Celite-adsorbed extract mixture. The mixture was loaded onto 20 g of prepacked C_18_ Sepak resin. The extract was fractionated by elution with a step gradient solvent system composed of MeOH and H_2_O (20%, 40%, 60%, 80%, and 100% MeOH in H_2_O). Depsidomycins B and C (**1** and **2**) eluted in the 80% aqueous MeOH fraction. To obtain pure compounds, further purification was performed using an analytical reverse-phase HPLC system (Phenomenex, Torrance, CA, USA) Luna 10 μm C_8_ (2) 250 × 4.6 mm, 35% to 70% aqueous CH_3_CN as the solvent gradient system over 40 min, flow rate of 1 mL/min, and UV detection at 210 nm). Depsidomycin B (**1**, 23.6 mg) and C (**2**, 7.3 mg) were collected at retention times of 28 min and 25 min, respectively.

*Depsidomycin B* (**1**): white powder; ${\left[\alpha \right]}_{D}^{25}$ −1.7 (c 0.2, MeOH); UV (MeOH) λ_max_ (log ε) 201 (2.31) nm; IR (neat) λ_max_ 3407, 2843, 1646, 1451, 1017 cm^−1^; ^1^H and ^13^C NMR data, see Table 1; HR-ESI-MS *m*/*z* 814.4799 [M + Na]^+^ (calcd for C_38_H_65_N_9_O_9_Na, 814.4798).

*Depsidomycin C* (**2**): white powder; ${\left[\alpha \right]}_{D}^{25}$ −0.01 (c 0.2, MeOH); UV (MeOH) λ_max_ (log ε) 200 (2.29) nm; IR (neat) λ_max_ 3375, 2948, 1657, 1451, 1024 cm^−1^; ^1^H and ^13^C NMR data, see Table 1; HR-ESI-MS *m*/*z* 800.4640 [M + Na]^+^ (calcd for C_37_H_63_N_9_O_9_Na, 800.4641).

### 3.5. Determination of the Absolute Configurations of the α-Carbons of the Amino Acid Residues

Depsidomycin B (1 mg) was hydrolyzed in 0.5 mL of 6 N HCl at 115 °C. After 90 min, the reaction vial was cooled to 0 °C in ice for 3 min. Then, the HCl in the reaction vial was removed in vacuo. The dry material was dissolved again in 1 mL of H_2_O and dried in vacuo. This process was repeated three times to completely remove the HCl. The hydrolysate was divided into two portions, and each portion was transferred to a 4-mL vial. To each vial, 100 μL of 1 N NaHCO_3_ was added to dissolve the hydrolysate. Then, 50 μL of either 10 mg/mL l-FDAA (l-fluoro-2,4-dinitrophenyl-5-l-alanine amide) or d-FDAA in acetone was added to the vials. After the reaction was maintained at 80 °C in a water bath for 3 min, 50 μL of 2 N HCl was added to quench the reaction by neutralization. Additionally, 300 μL of 50% aqueous CH_3_CN was added to the solution to dissolve the reaction products. A 10-μL aliquot of each reaction mixture was analyzed by LC/MS with a gradient solvent system (20% to 60% CH_3_CN with 0.1% formic acid over 40 min) on a C_18_ reversed-phase column (100 × 4.6 mm) with UV detection at 210 nm (Table S4). The absolute configurations of the amino acid residues in depsidomycin C (**2**) were determined using the procedure described above.

### 3.6. Determination of the Absolute Configurations at the β-Carbons of Threonine and Isoleucine

The hydrolysates of **1** and **2** were dissolved in H_2_O (1 mg/mL) in two separate 4-mL vials. Then, 100 μL of 1% GITC (2,3,4,6-tetra-*O*-acetyl-β-d-glucopyranosyl isothiocyanate) in acetone and 100 μL of 6% triethylamine were added to each vial. Each reaction mixture was stirred for 15 min at rt, and then 100 μL of 5% acetic acid was added to dilute the reaction mixtures. The products were analyzed by LC/MS under different gradient solvent systems (10% to 100% CH_3_CN with 0.1% formic acid over 20 min on a Phenomenex Luna C_18_ reversed-phase column, 100 × 4.6 mm, for Thr, and 30% to 55% CH_3_CN with 0.1% formic acid over 80 min on a Phenomenex Luna C_18_ reversed-phase column, 250 × 4.6 mm, for Ile). Detection at 210 nm was performed by a UV detector. The authentic standards of d-Thr, d-*allo*-Thr, d-Ile and d-*allo*-Ile were also derivatized with GITC by using the identical method. The GITC-derivatized products for d-Thr in **1** and **2** eluted at 9.4 min, indicating that the threonine units in **1** and **2** are d-Thr and not d-*allo*-Thr. The GITC adduct of d-Ile in **1** was detected at 53.7 min, which establish that this residue is d-Ile and not d-*allo*-Ile. Then, the GITC derivatives of the hydrolysate were co-injected with the GITC derivatives of each of the standards (d-Thr, d-*allo*-Thr, d-Ile and d-*allo*-Ile) to confirm the assignments. The GITC derivatives of authentic d-Thr, d-*allo*-Thr, d-Ile and d-*allo*-Ile eluted at 9.4, 9.6, 53.7 and 52.3 min, respectively, confirming the configurations of the β-carbons of d-Thr and d-Ile.

### 3.7. Methanolysis of Depsidomycin B *(**1**)*

Depsidomycin B (**1**, 8 mg) was dissolved in 2 mL of MeOH, and 11.2 mg of NaOMe was added to the vial to prepare a 0.5 M NaOMe solution. The reaction mixture was stirred at r.t. for 3 h, and the reaction was quenched with 1 N HCl. After evaporating the MeOH in vacuo, the residue of the methanolysis reaction was partitioned into H_2_O and EtOAc. After removing the solvent, the methanolysis products (**3**, 6 mg) were purified on a Sephadex LH-20 (GE Healthcare, Chicago, IL, USA) open column using MeOH as the eluent. The structure of the methanolysis product (**3**) was assigned by analyzing its 1D and 2D NMR and LC-MS/MS data. For ^1^H and ^13^C NMR data, see Table S2, HR-ESI-MS *m*/*z* 824.5252 [M + H]^+^ (calcd. for C_39_H_70_N_9_O_10_ 824.5245).

### 3.8. Evaluation of Antimetatastic Activity

MDA-MB-231 cells were used to measure the antimetastatic activities of **1** and **2** based on the wound-healing assay method described previously in the literature [18]. Sunitinib was used as a positive control (Figure S33).

### 3.9. Statistical Analysis

Data are expressed as the mean ± standard deviation (SD) for the indicated number of independently performed experiments. Statistical significance was analyzed using a Student’s *t*-test or one-way analysis of variance (ANOVA) coupled with a Dunnett’s *t*-test. Differences were considered statistically significant at * *p* < 0.05, ** *p* < 0.01.

## 4. Conclusions

Depsidomycins B and C (**1** and **2**) are new cyclic heptapeptides bearing modified amino acid residues including two *S*-piperazic acids and d-Leu, d-Val, d-Thr, and d-Ile (d-Val for **2**), along with formyl groups at their *N*-termini. Even though the planar structure of depsidomycin B (**1**) is identical to that of the previously reported compound depsidomycin, the absolute configurations of the amino acids in **1** are clearly different from those in depsidomycin [10]. In addition, considering that the piperazic acid units in the original literature on depsidomycin were not configurationally assigned [10], this work constitutes the first report of new members of the depsidomycin class of cyclic peptides and the first complete stereochemical structure elucidation of these compounds. The discovery of these new compounds from a *Streptomyces* sp. strain isolated from ginseng farm soil based on a time-course LC/MS analysis of its metabolite profiles suggests that the investigation of farm soil-derived actinomycetes using a chemical analysis-based search strategy could provide an additional route for the effective discovery of new bioactive natural products.

## Figures and Tables

**Table 1:** ^1^H and ^13^C NMR data of depsidomycins B and C (**1** and **2**) in acetone-*d*_6_ *^a^*.

Position	Depsidomycin B (1)		Depsidomycin C (2)	
	δ_C_, Type	δ_H_, mult (*J* in Hz) *^a^*	δ_C_, Type	δ_H_, mult (*J* in Hz) *^a^*
1	169.3, C		170.0, C	
2	51.4, CH	5.18, br s	51.5, CH	5.17, br s
3a	23.4, CH_2_	1.85, m	23.3, CH_2_	1.87, m
3b		2.19, m		2.19, m
4a	21.3, CH_2_	1.55, m	21.9, CH_2_	1.55, m
4b		1.86, m		
5a	47.9, CH_2_	3.12, m	48.4, CH_2_	3.15, m
5b		2.85, m		2.85, m
5-NH		4.86, m		4.85, m
6	175.4, C		175.9, C	
7	49.7, CH	5.40, dd (10.5, 10.5)	49.4, CH	5.39, dd (10.5, 10.5)
7-NH		7.62, br d (10.5)		7.60, br d (10.5)
8a	41.4, CH_2_	1.68, m	41.9, CH_2_	1.68, m
8b		1.95, m		1.97, m
9	26.6, CH	1.78, m	26.7, CH	1.78, m
10	21.0, CH_3_	1.02, d (6.5)	21.9, CH_3_	1.03, d (6.5)
11	23.7, CH_3_	0.93, d (6.5)	19.7, CH_3_	0.96, d (6.5)
12	167.7, C		168.5, C	
13	52.8, CH	5.12, d (4.5)	53.1, CH	5.11, d (4.5)
14a	24.9, CH_2_	1.58, m	25.5, CH_2_	1.58, m
14b		2.57, br d (13.5)		2.57, br d (13.3)
15a	23.0, CH_2_	1.39, m	23.7, CH_2_	1.39, m
15b		1.55, br d (10.5)		1.55, br d (10.5)
16a	47.8, CH_2_	2.75, m	48.5, CH	2.77, m
16b		3.12, m		3.12, m
16-NH		3.95, d (12.5)		3.94, d (12.5)
17	175.9, C		175.9, C	
18	56.2, CH	5.20, dd (10.0, 6.0)	56.2, CH	5.20, dd (10.5, 6.0)
18-NH		7.59, d (6.0)		7.58, d(6.0)
19	29,5, CH	1.99, m	29.8, CH	1.99, m
20	21.0, CH_3_	1.02, d (6.5)	20.8, CH_3_	1.04, d (6.5)
21	19.7, CH_3_	0.97, d (6.5)	19.8, CH_3_	0.97, d (6.5)
22	174.7, C		175.7, C	
23	51.0, CH	4.88, m	51.7, CH	4.87, m
23-NH		7.07, br s		7.05, d (9.5)
24a 24b	40.8, CH_2_	1.68, m 2.17, m	41.6 CH_2_	1.69, m 2.17, m
25	25.8, CH	1.71, m	26.3, CH	1.70, m
26	20.6, CH_3_	0.89, m	20.7, CH_3_	0.89, m
27	24.0, CH_3_	0.91, m		0.90, m
28	167.4, C		168.1, C	
29	55.8, CH	4.48, br s	55.8, CH	4.47, m
29-NH		7.52, br s		7.50, d (6.0)
30	71.4, CH	4.91, m	72.0, CH	4.90, m
31	13.6, CH_3_	1.17, d (6.5)	13.6, CH_3_	1.17, d (6.5)
32	171.7, C		171.6, C	
33	55.4, CH	4.66, m	57.3, CH	4.49, m
33-NH		7.49, br s		7.49, br d (6.5)
34	38.1, CH	1.98, m	32.2, CH	2.18, m
35a	26.9, CH_2_	1.21, m	23.9, CH_3_	0.93, d (6.5)
35b		1.43, m		
36	11.8, CH_3_	0.90, m	24.0. CH_3_	0.92, d (6.5)
37	14.6, CH_3_	0.87, d (7.0)	161.7, CH	8.25, s
38	161.8, CH	8.27, s		

*^a^*^1^H and ^13^C NMR were recorded at 850 and 212.5 MHz, respectively.

## Supplementary Materials

The supplementary materials are available online.
