# Supplementary material for: Depsidomycins B and C: New Cyclic Peptides from a Ginseng Farm Soil-Derived Actinomycete
Source: Molecules. 2018 May 25;23(6):1266. doi: 10.3390/molecules23061266 (PMC6099933; doi:10.3390/molecules23061266)
Supplement: Supplementary file 1 [file molecules-23-01266-s001.pdf]

Supporting Information

Depsidomycins B and C: New Cyclic Peptides from a Ginseng Farm Soil-derived Actinomycete.

Yun Kwon [1], Woong Sub Byun [1], Byung-Yong Kim [2], Myoung Chong Song [3], Munhyung Bae [1], Yeo Joon Yoon [3], Jongheon Shin [1], Sang Kook Lee [1] and Dong-Chan Oh [1,*]

[1] Natural Product Research Institute, College of Pharmacy, Seoul National University, Seoul 08826, Republic of Korea
[2] ChunLab, Inc., JW TOWER, Seocho-gu, Seoul 06725, Republic of Korea
[3] Department of Chemistry and Nanoscience, Ewha Womans University, Seoul 03760, Republic of Korea
* Correspondence: dongchanoh@snu.ac.kr; Tel.: +82-288-024-91

Table of Contents

Figure S1. ^{1}H NMR spectrum (850 MHz) of depsidomycin B (**1**) in pyridine–*d*5.

Figure S2. ^{13}C NMR spectrum (212.5 MHz) of **1** in pyridine–*d*5.

Figure S3. COSY spectrum (850 MHz) of **1** in pyridine–*d*5.

Figure S4. HSQC spectrum (850 MHz) of **1** in pyridine–*d*5.

Figure S5. HMBC spectrum (850 MHz) of **1** in pyridine–*d*5.

Figure S6. ROESY spectrum (850 MHz) of **1** in pyridine–*d*5.

Figure S7. TOCSY spectrum (850 MHz) of **1** in pyridine–*d*5

Figure S8. ^{1}H NMR spectrum (850 MHz) of **1** in acetone–*d*6.

Figure S9. ^{13}C NMR spectrum (212.5 MHz) of **1** in acetone–*d*6.

Figure S10. COSY spectrum (850 MHz) of **1** in acetone–*d*6.

Figure S11. HSQC spectrum (850 MHz) of **1** in acetone–*d*6.

Figure S12. HMBC spectrum (850 MHz) of **1** in acetone–*d*6.

Figure S13. ROESY spectrum (850 MHz) of **1** in acetone–*d*6.

Figure S14. TOCSY spectrum (850 MHz) of **1** in acetone–*d*6.

Figure S15. ^{1}H NMR spectrum (850 MHz) of depsidomycin C (**2**) in acetone–*d*6.

Figure S16. ^{13}C NMR spectrum (212.5 MHz) of **2** in acetone–*d*6.

Figure S17. COSY spectrum (850 MHz) of **2** in acetone–*d*6.

Figure S18. HSQC spectrum (850 MHz) of **2** in acetone–*d*6.

Figure S19. HMBC spectrum (850 MHz) of **2** in acetone–*d*6.

Figure S20. ROESY spectrum (850 MHz) of **2** in acetone–*d*6.

Figure S21. TOCSY spectrum (850 MHz) of **2** in acetone–*d*6.

Figure S22. ^{1}H NMR spectrum (600 MHz) of methanolysis product (**3**) of **1** in acetone–*d*6.

Figure S23. ^{13}C NMR spectrum (150 MHz) of **3** in acetone–*d*6.

Figure S24. COSY spectrum (600 MHz) of **3** in acetone–*d*6.

Figure S25. HSQC spectrum (600 MHz) of **3** in acetone–*d*6.

Figure S26. HMBC spectrum (600 MHz) of **3** in acetone–*d*6.

Figure S27. TOCSY spectrum (600 MHz) of **3** in acetone–d_6.

Figure S29. LC/MS chromatogram of (a) L- and (b) D-FDAA derivatives of **1**.

Figure S30. LC/MS chromatogram of (a) L- and (b) D-FDAA derivatives of **2**.

Figure S31. LC/MS chromatogram of GITC derivatives of (a) **1**, (b) authentic D-Ile, (c) D-*allo*-Ile, (d) coinjection of GITC derivatives of **1** with authentic (d) D-Ile and (e) D-*allo*-Ile.

Figure S32. LC/MS chromatogram of GITC derivatives of (a) **1**, (b) authentic D-Thr, (c) L-Thr, (d) D-*allo*- and L-*allo*-Thr, and coinjection of GITC derivatives of authentic D-Thr with those of (e) **1** and (f) **2**.

Figure S33. Positive control data of wound healing assay using sunitinib.

Figure S34. Time-course LC/MS analysis of the culture of the strain BYK1371. Depsidomycins B and C (**1**-**2**) were detected at 15.4 and 14.6 min, respectively.

Figure S35. Phylogenetic relationships of single strain BYK 1371 and related S*treptomyces* taxa.

Table S1. ^{1}H and ^{13}C NMR data for **1** in pyridine-d_5.

Table S2. ^{1}H and ^{13}C NMR data for **3** in acetone-d_6.

Table S3. ^{1}H and ^{13}C NMR data for depsidomycin and B (**1**) in acetone-d_6.

Table S4. LC/MS analysis of the FDAA derivatives of **1** and **2**.

References

Figure S1. ^{1}H NMR spectrum (850 MHz) of **1** in pyridine–*d*5.

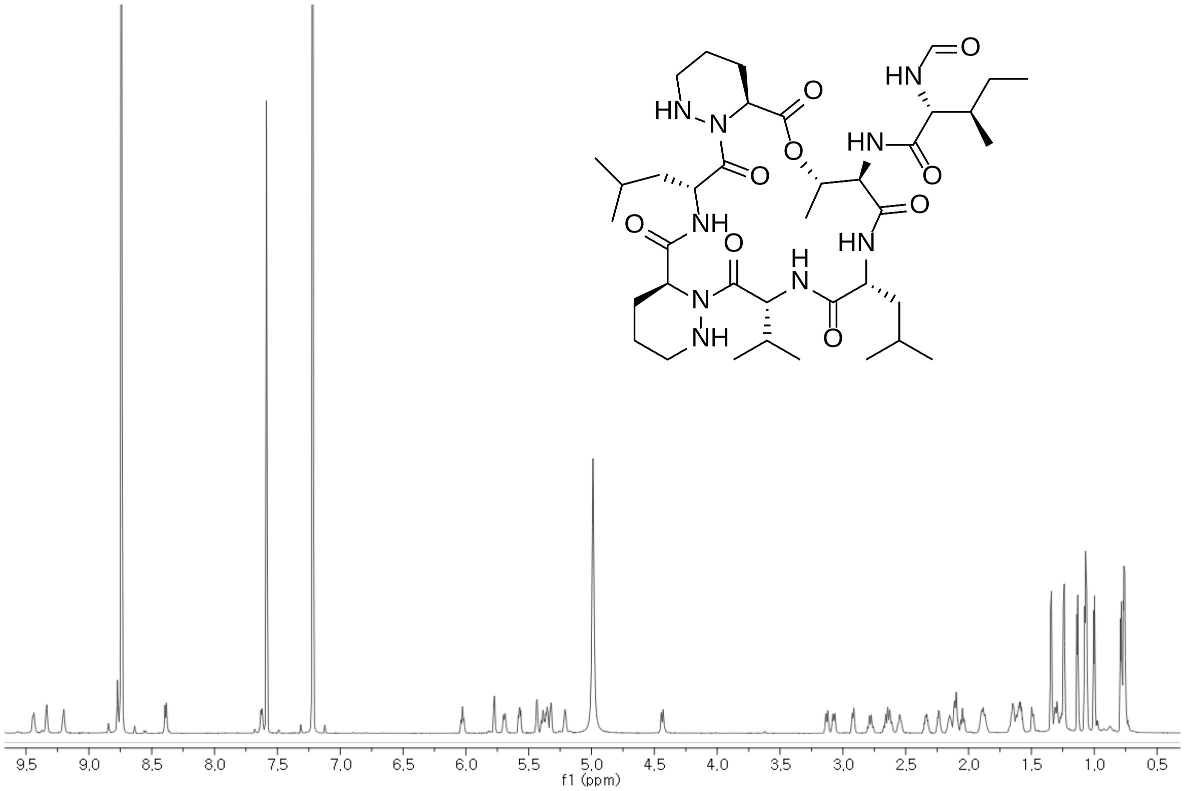

Figure S2. ^{13}C NMR spectrum (212.5 MHz) of **1** in pyridine–d_5.

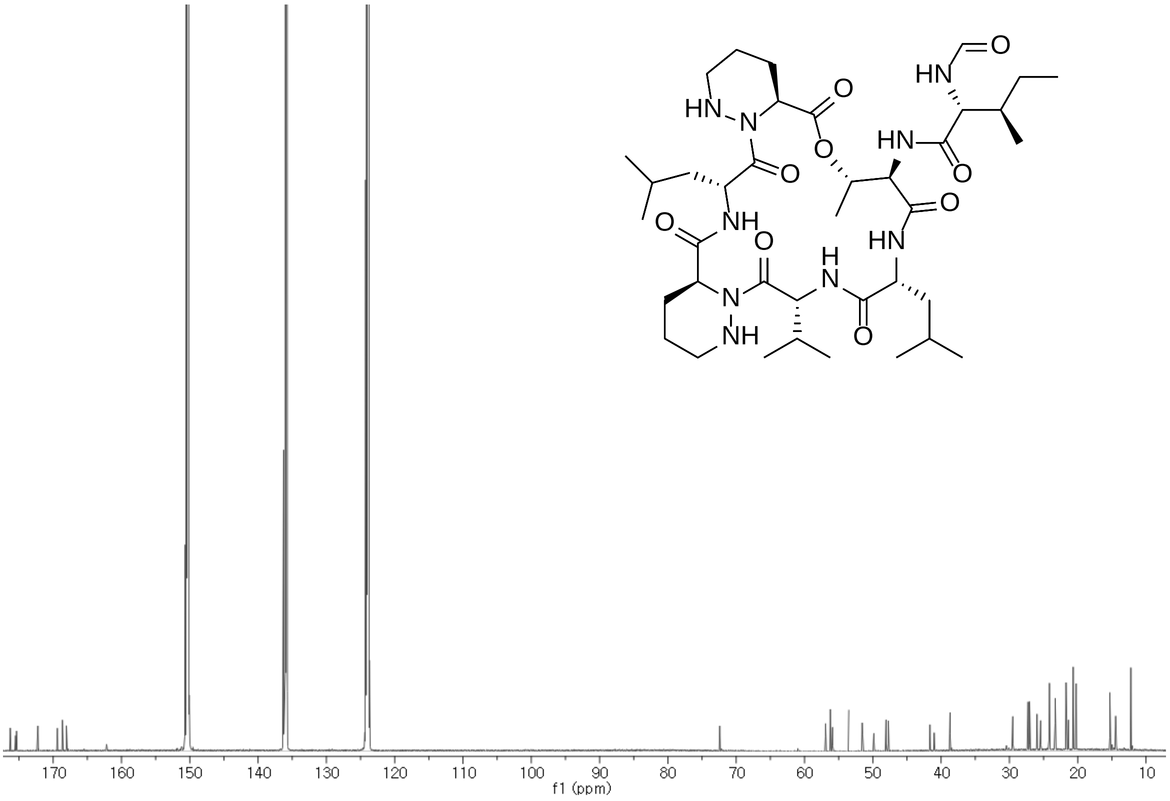

Figure S3. COSY spectrum (850 MHz) of **1** in pyridine–d_5.

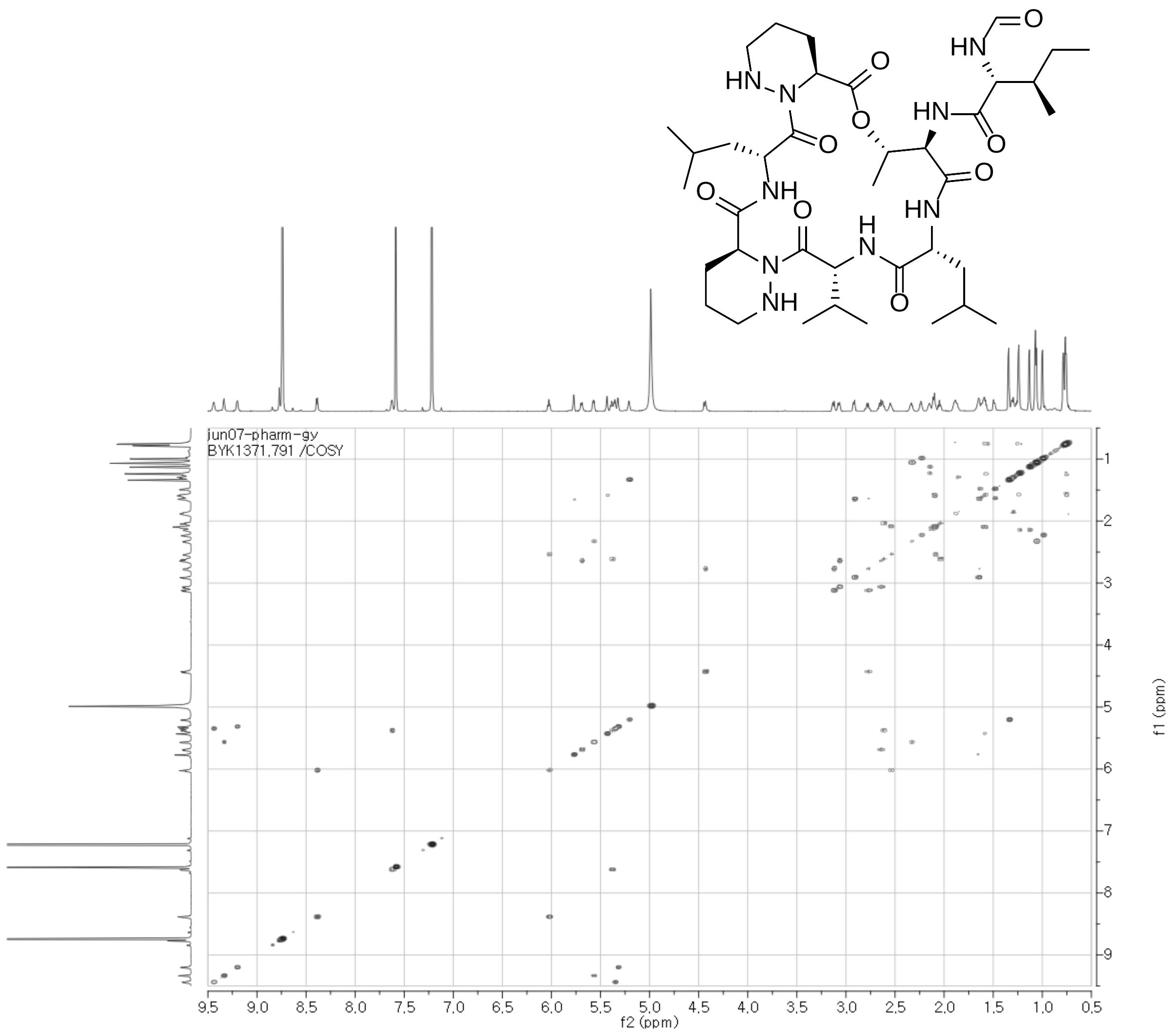

Figure S4. HSQC spectrum (850 MHz) of **1** in pyridine–d_5.

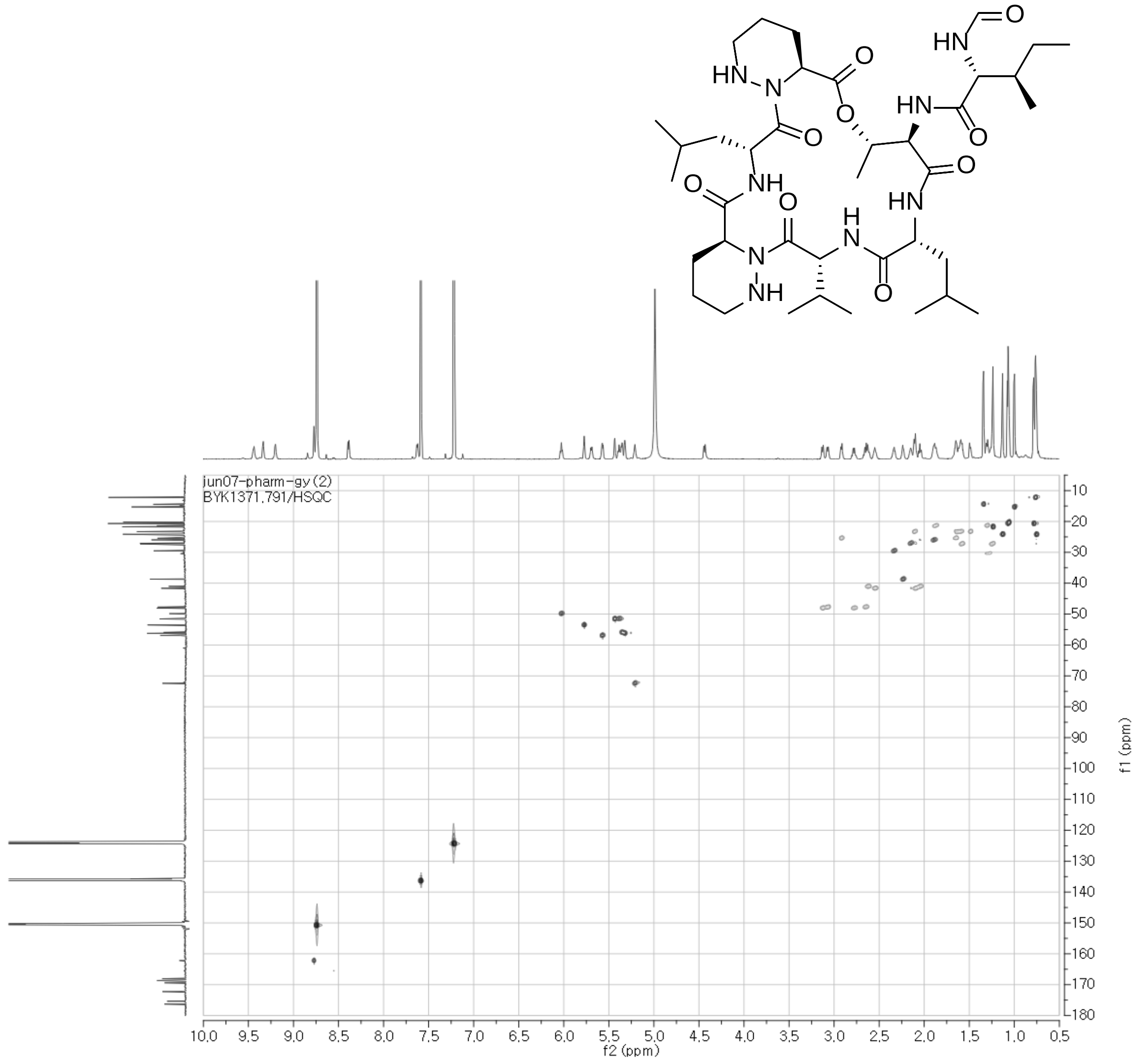

Figure S5. HMBC spectrum (850 MHz) of **1** in pyridine–d_5.

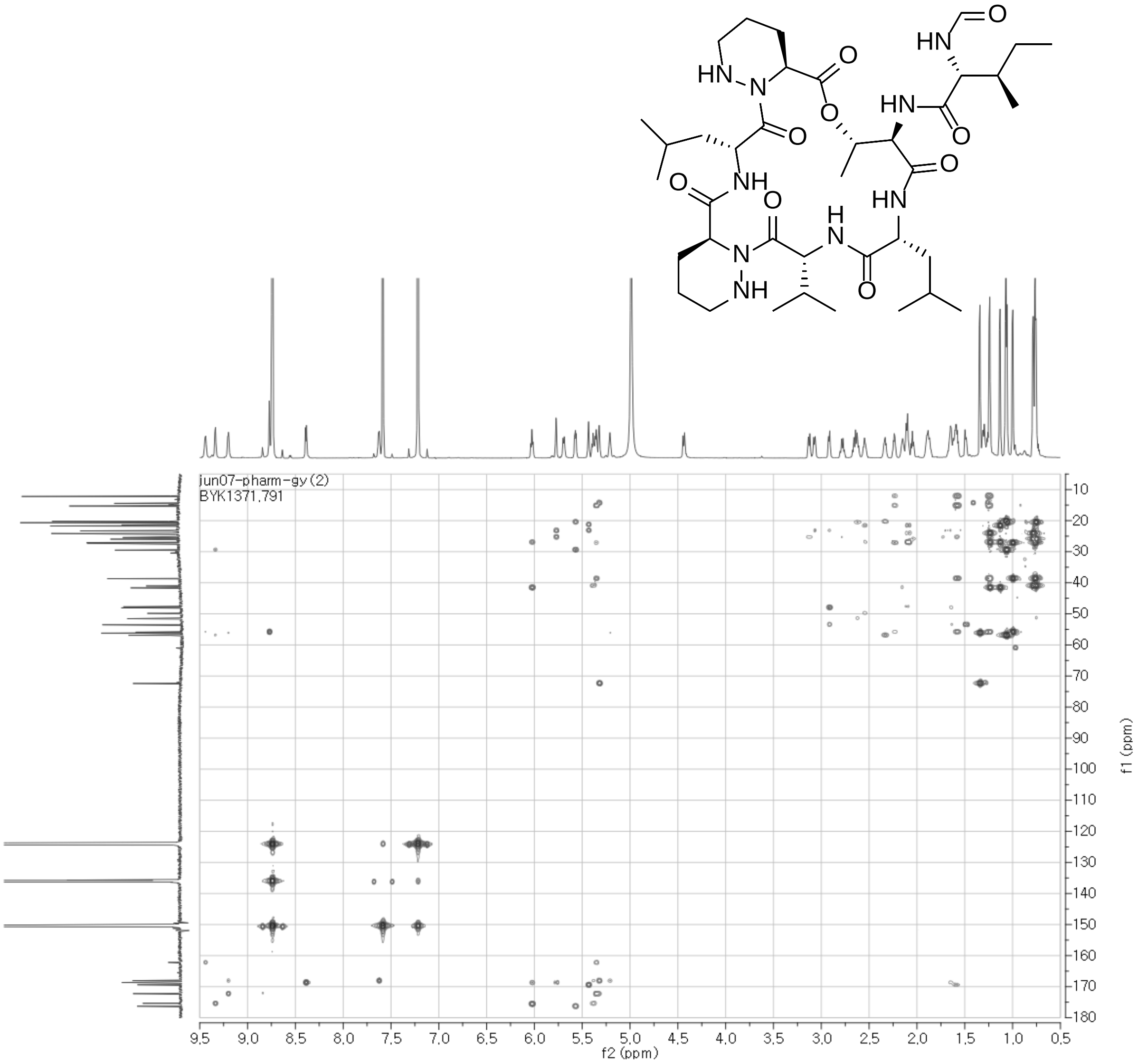

Figure S6. ROESY spectrum (850 MHz) of **1** in pyridine–d_5.

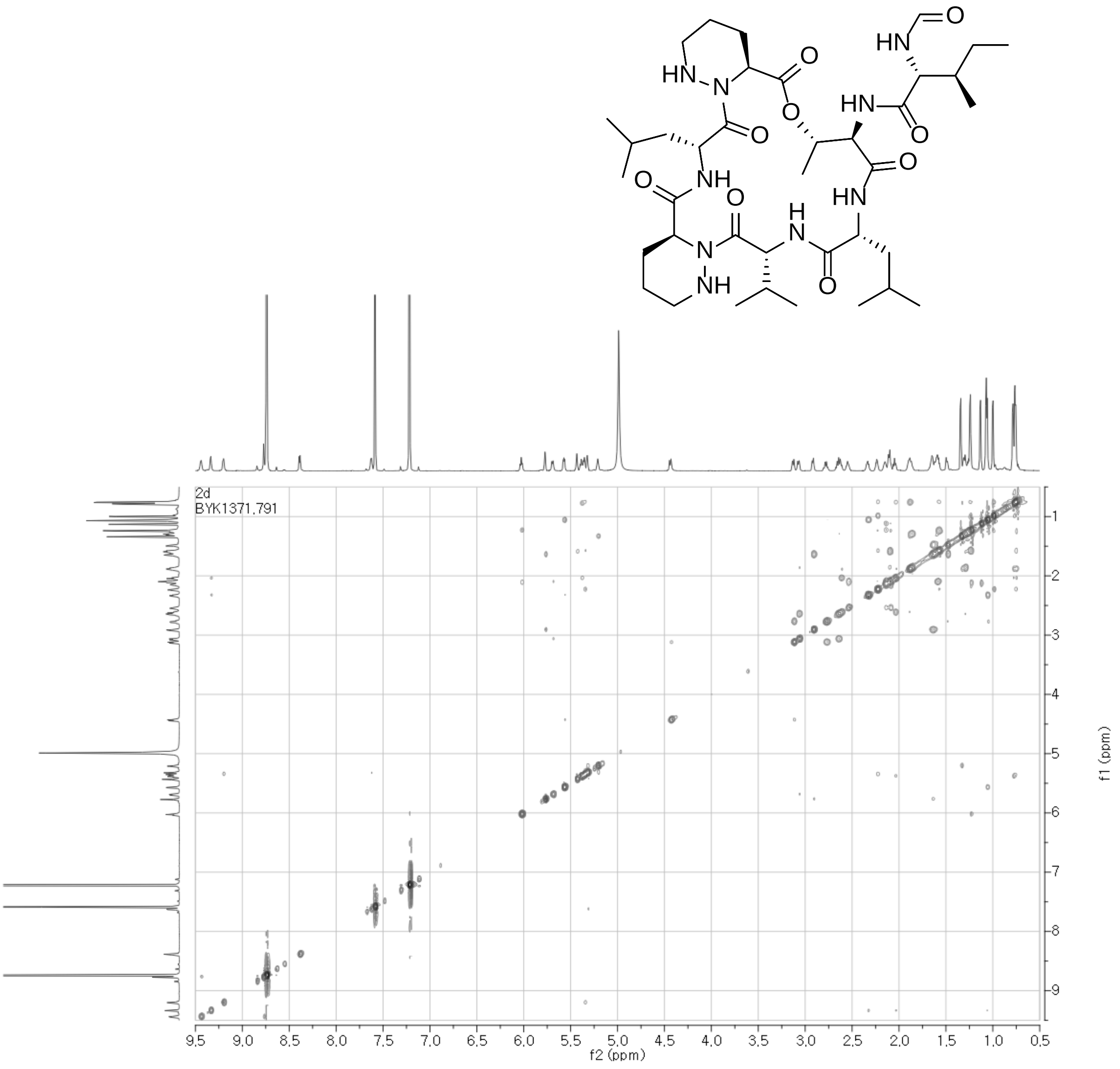

Figure S7. TOCSY spectrum (850 MHz) of **1** in pyridine–d_5.

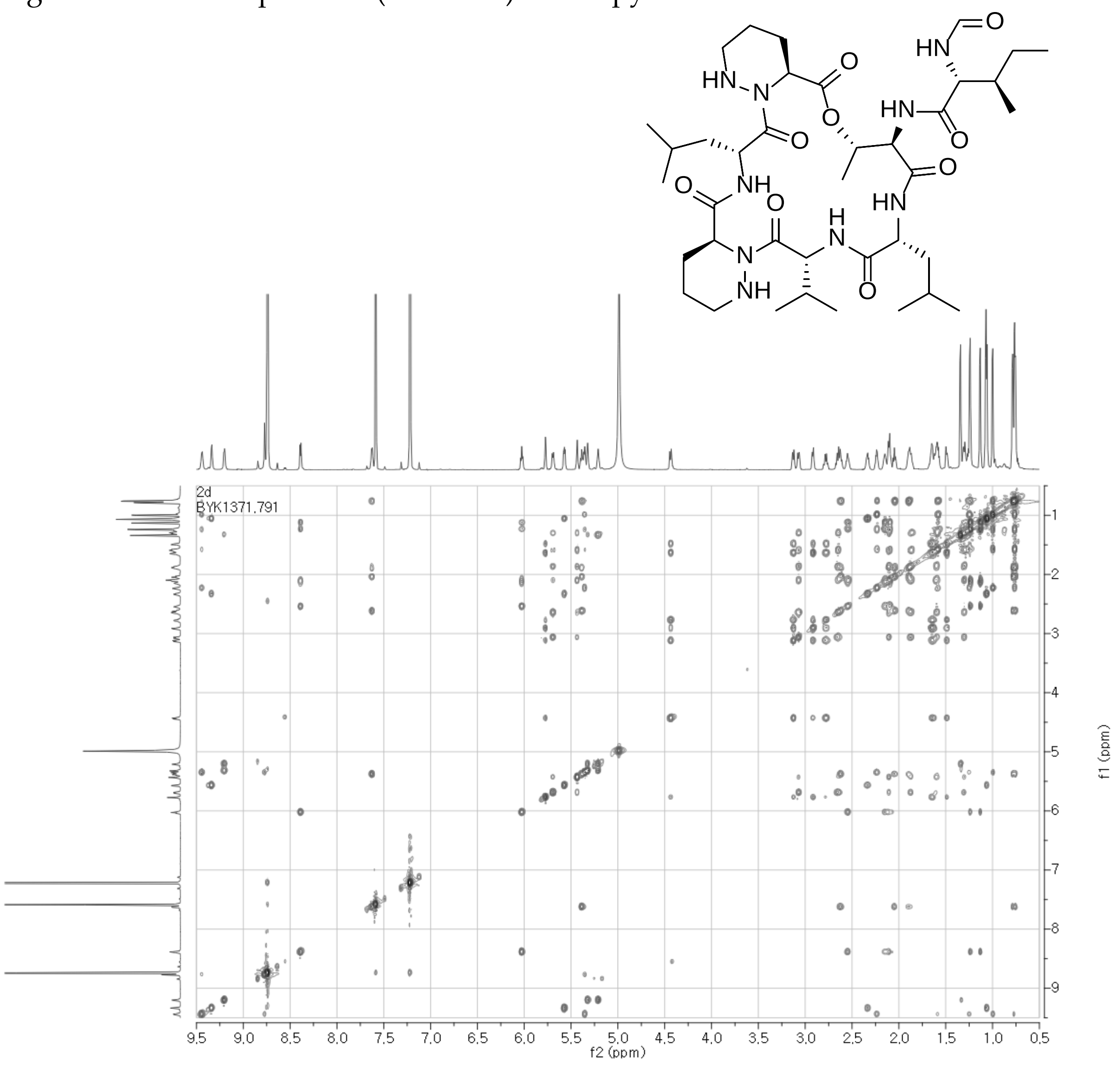

Figure S8. ^{1}H NMR spectrum (850 MHz) of **1** in acetone–d_6.

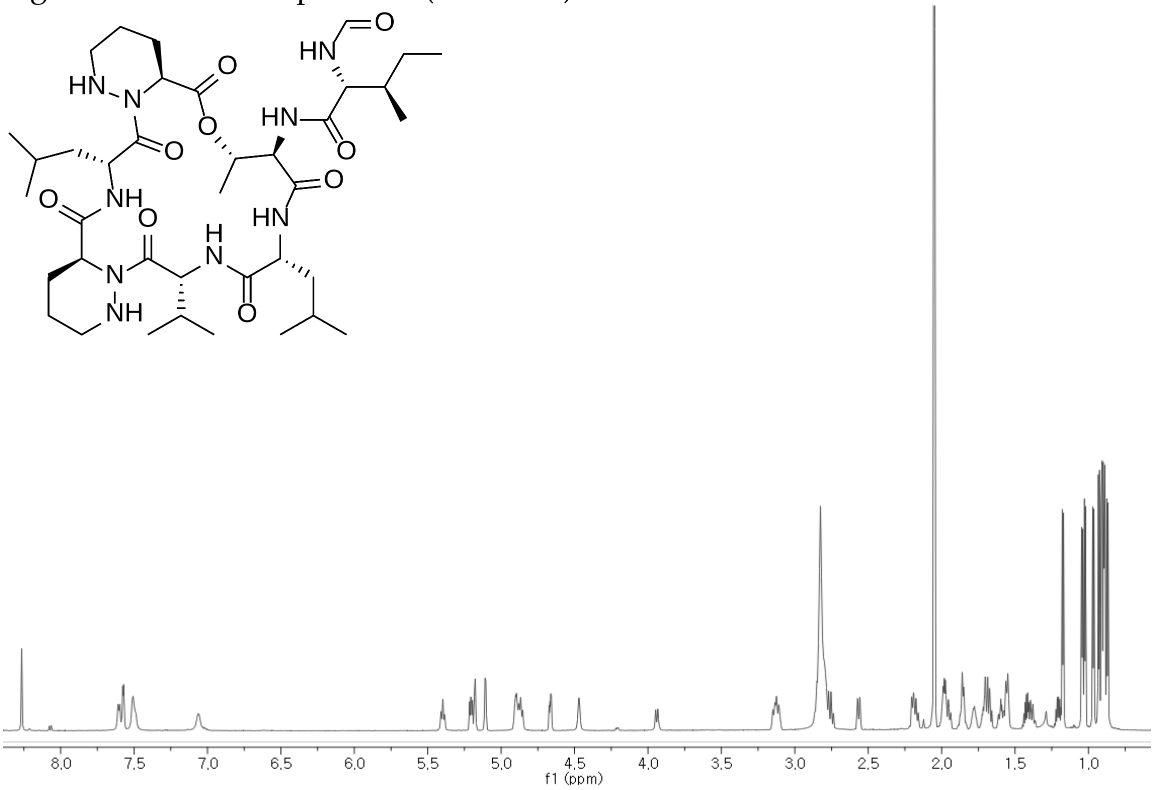

Figure S9. ^{13}C NMR spectrum (212.5 MHz) of **1** in acetone–d_6.

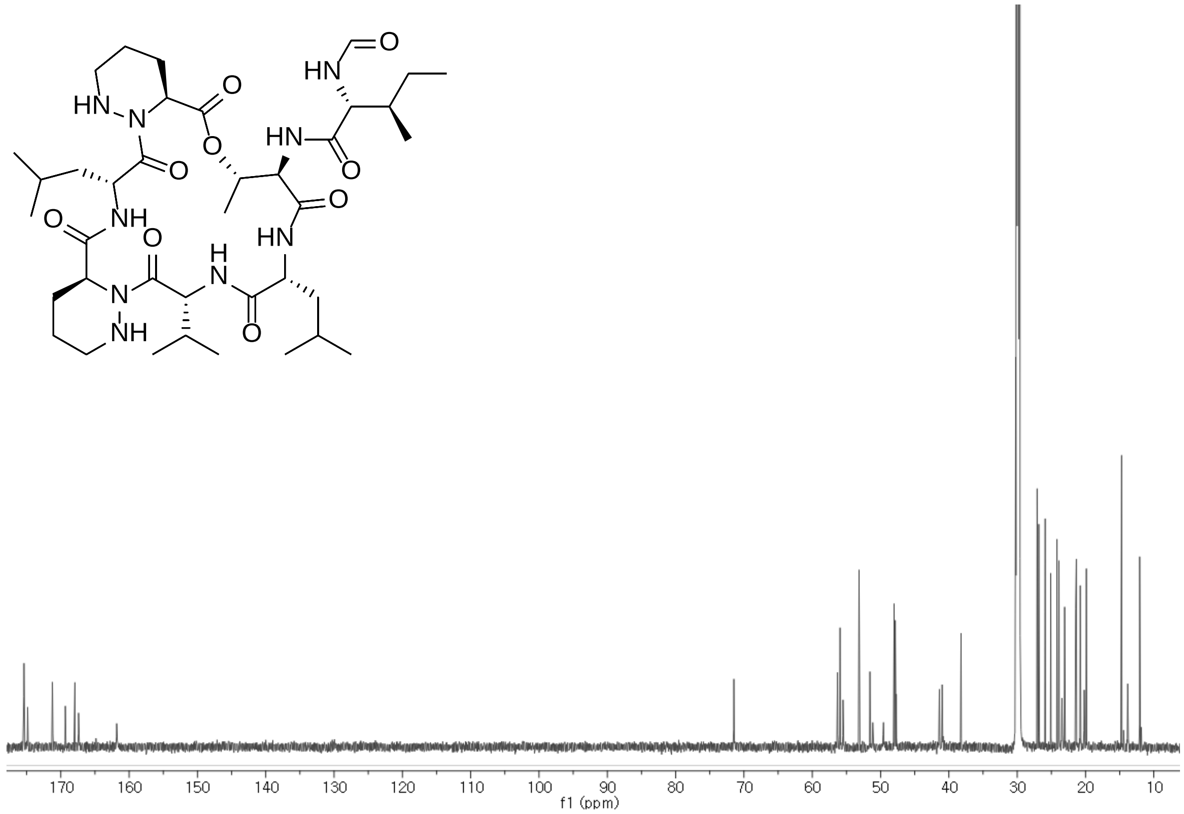

Figure S10. COSY spectrum (850 MHz) of **1** in acetone–d_6.

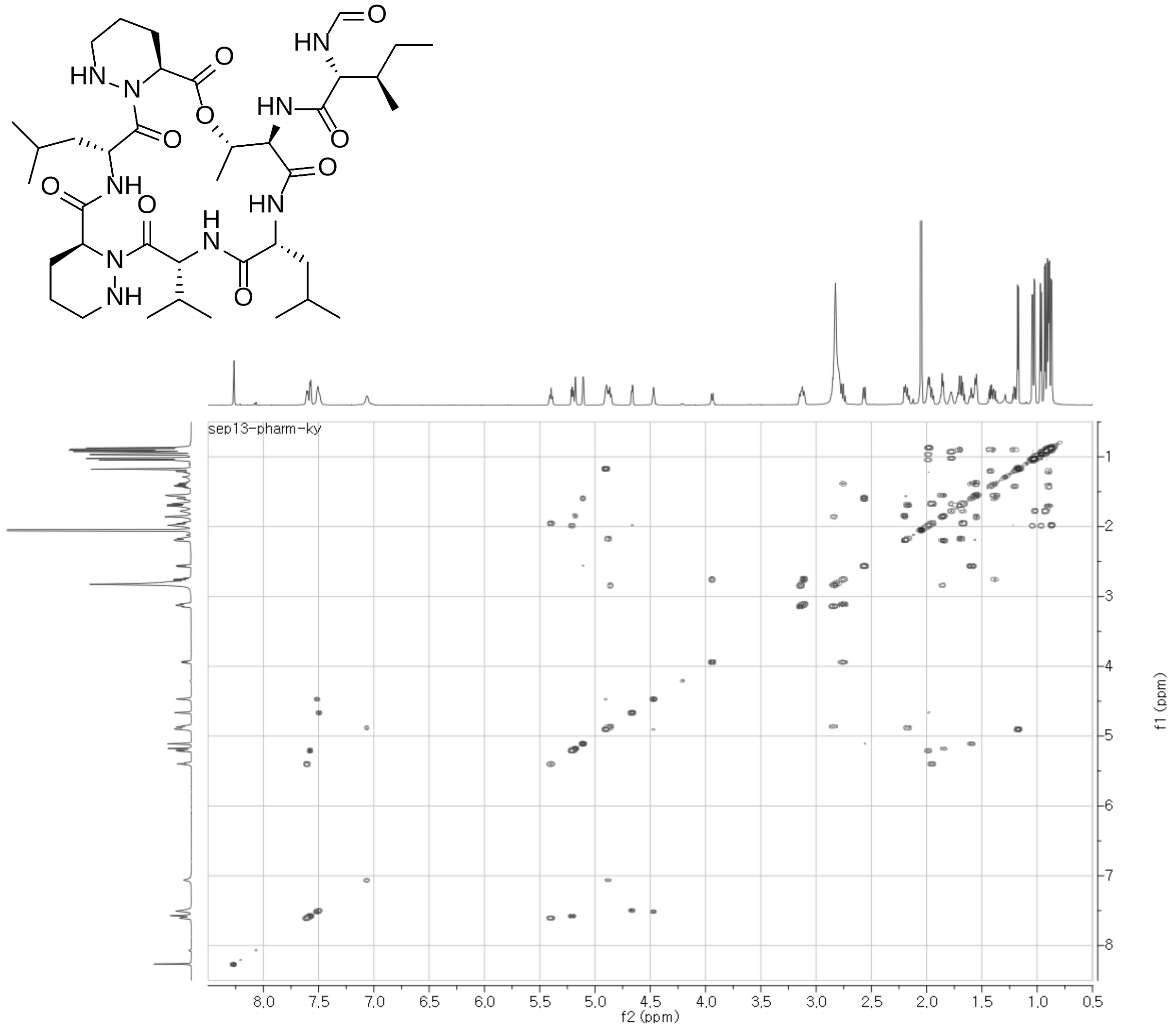

Figure S11. HSQC spectrum (850 MHz) of **1** in acetone–d_6.

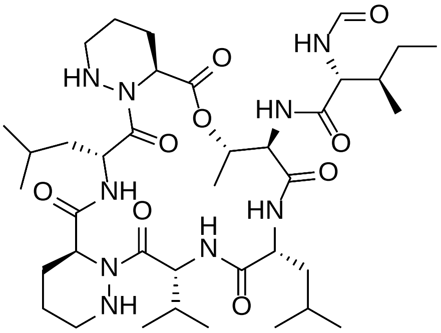

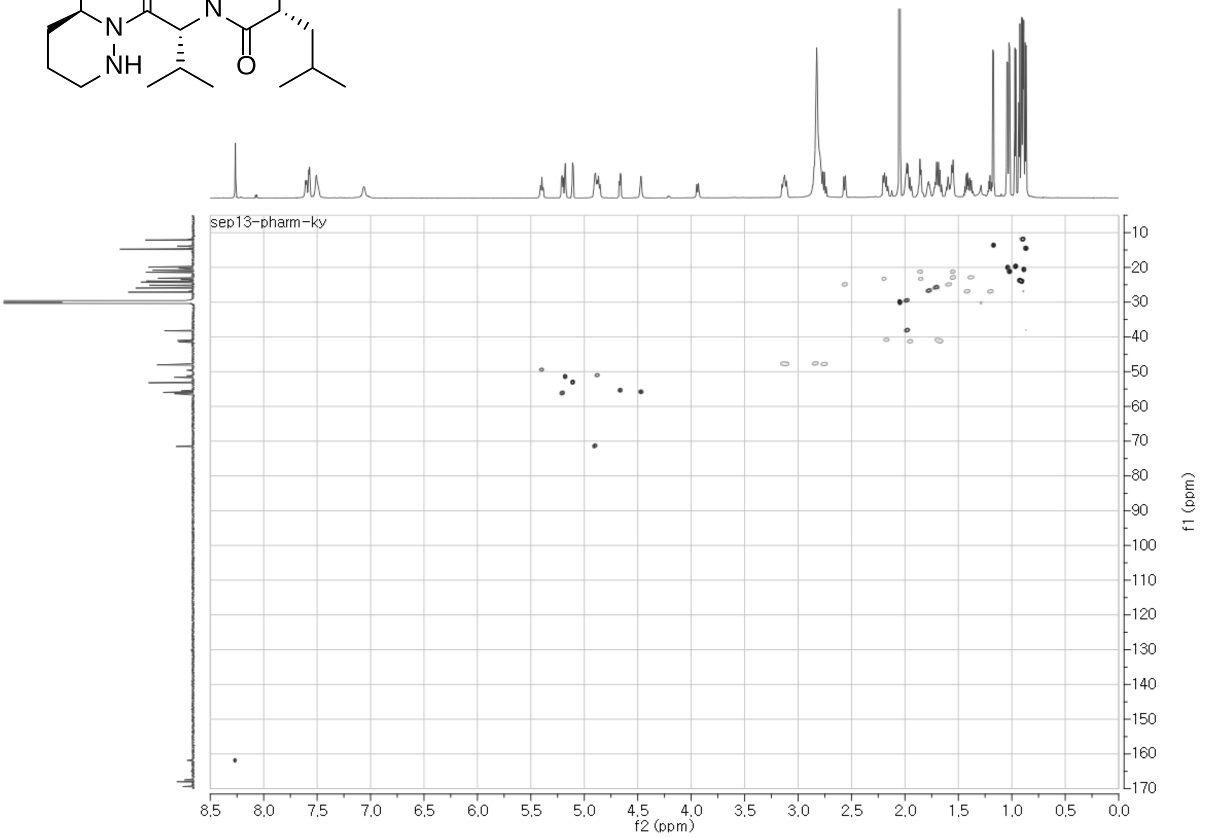

Figure S12. HMBC spectrum (850 MHz) of **1** in acetone–d_6.

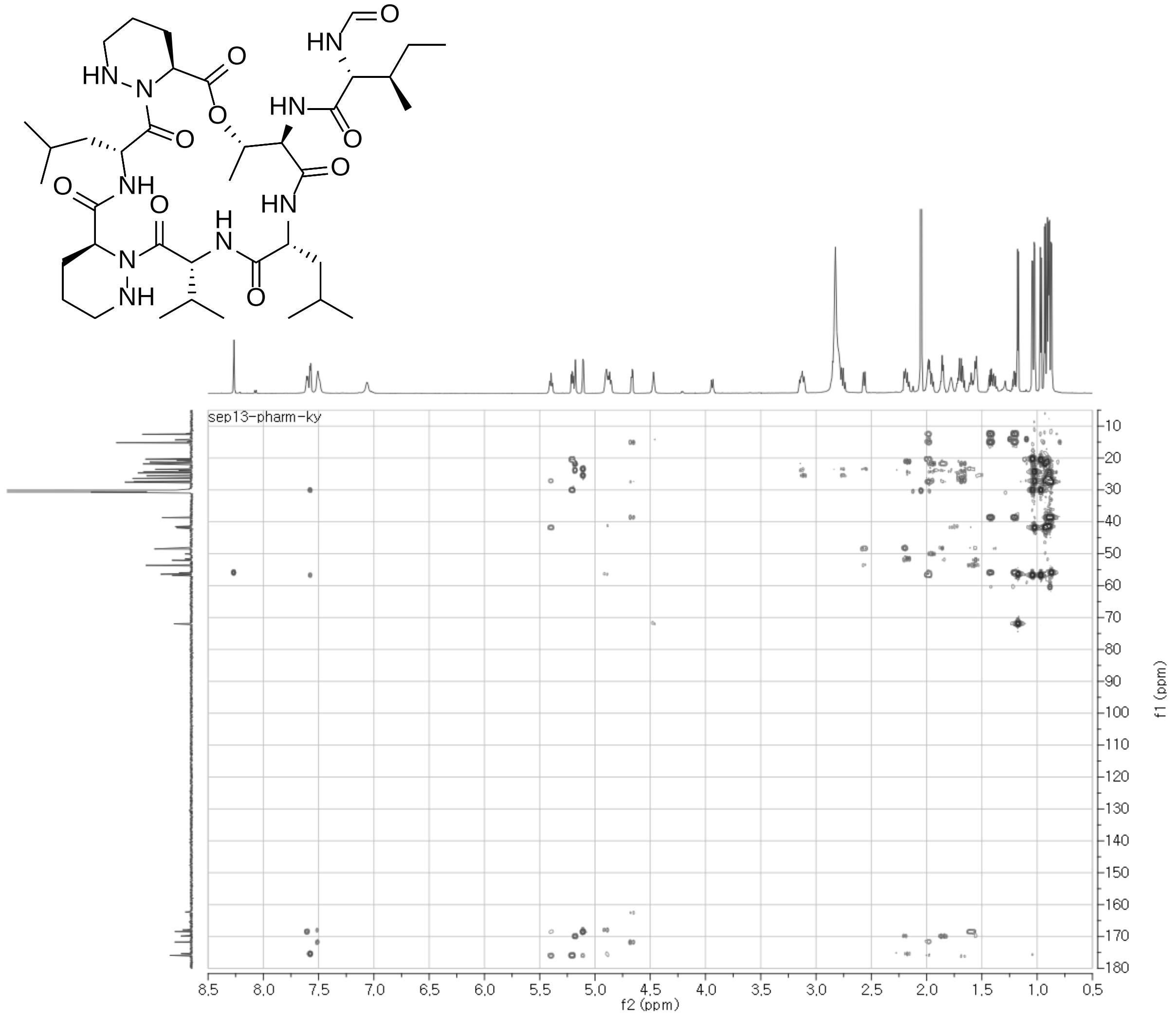

Figure S13. ROESY spectrum (850 MHz) of **1** in acetone–d_6.

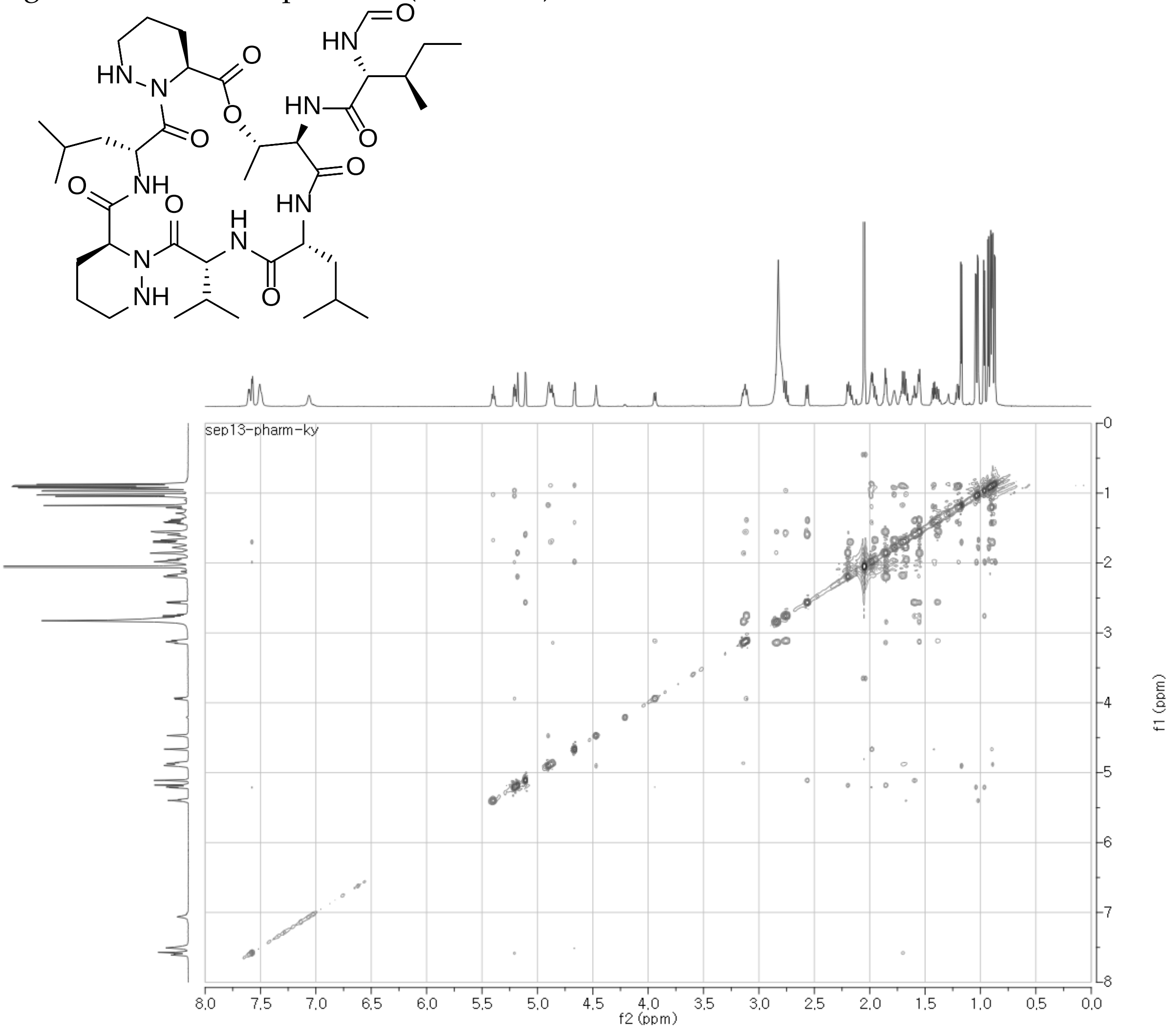

Figure S14. TOCSY spectrum (850 MHz) of **1** in acetone–d_6.

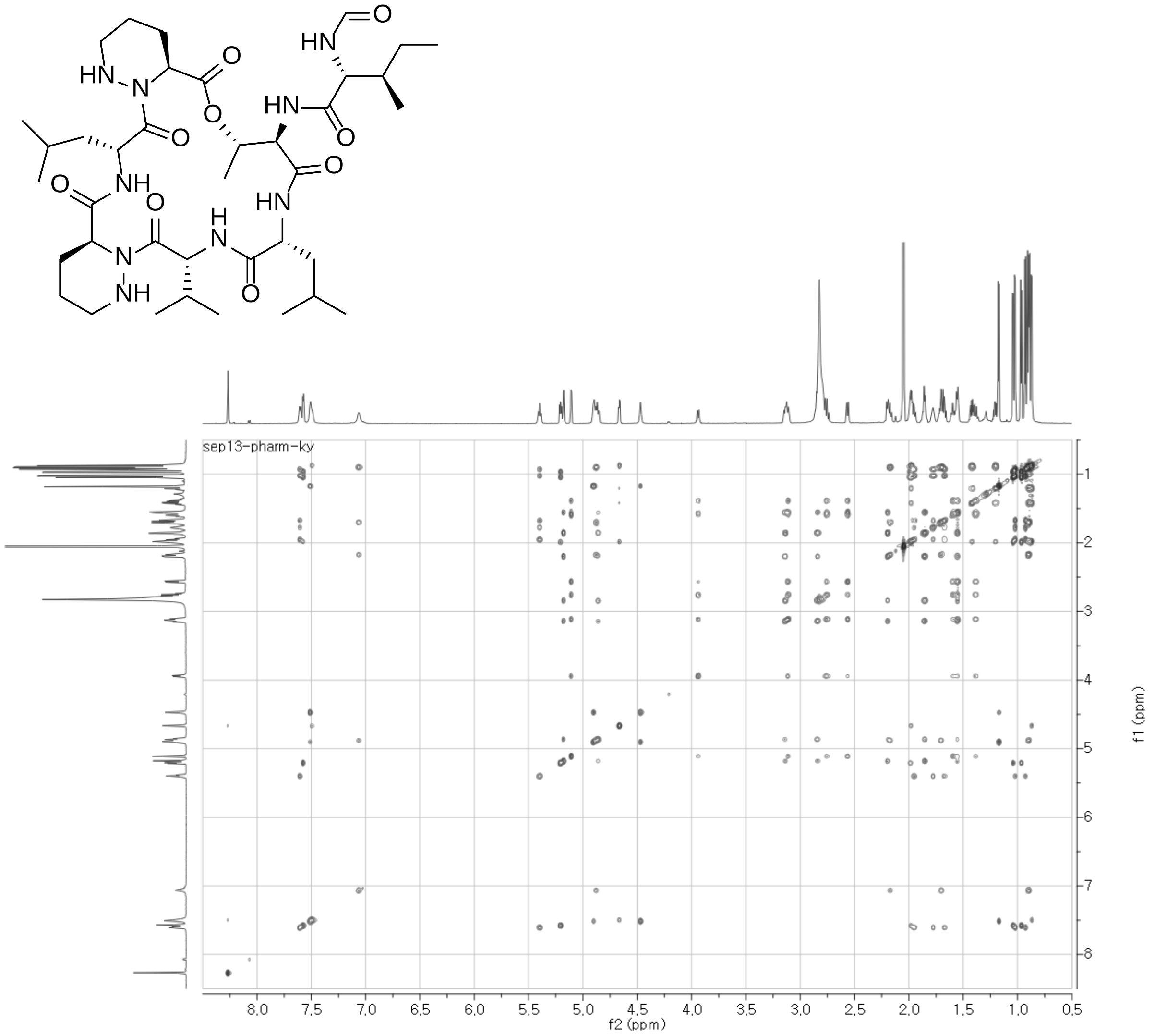

Figure S15. ^{1}H NMR spectrum (850 MHz) of **2** in acetone–d_6.

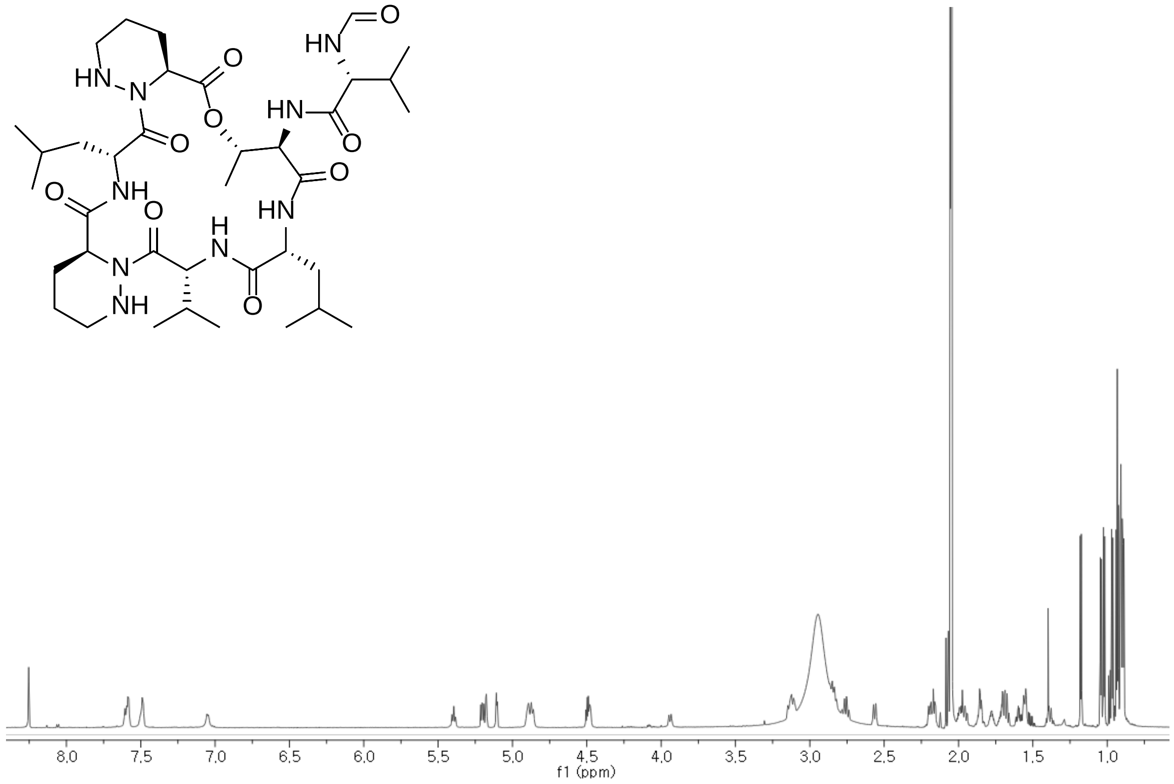

Figure S16. ^{13}C NMR spectrum (212.5 MHz) of **2** in acetone–d_6.

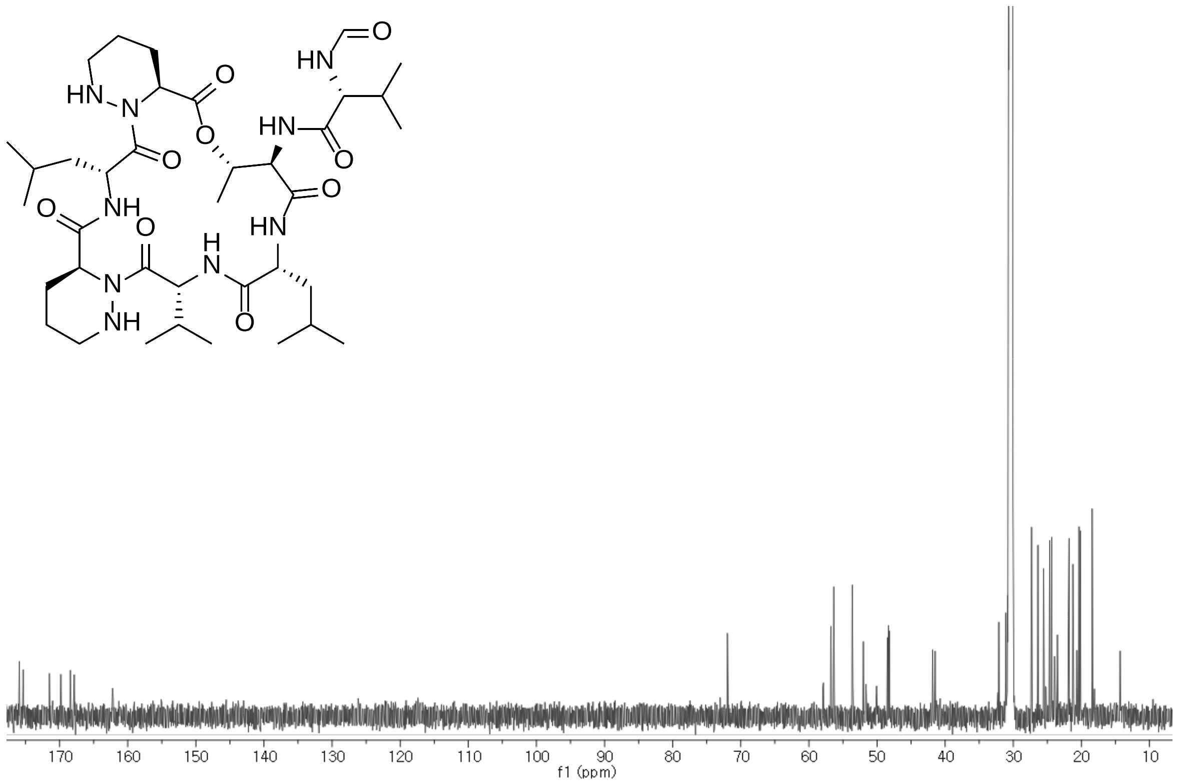

Figure S17. COSY spectrum (850 MHz) of **2** in acetone–d_6.

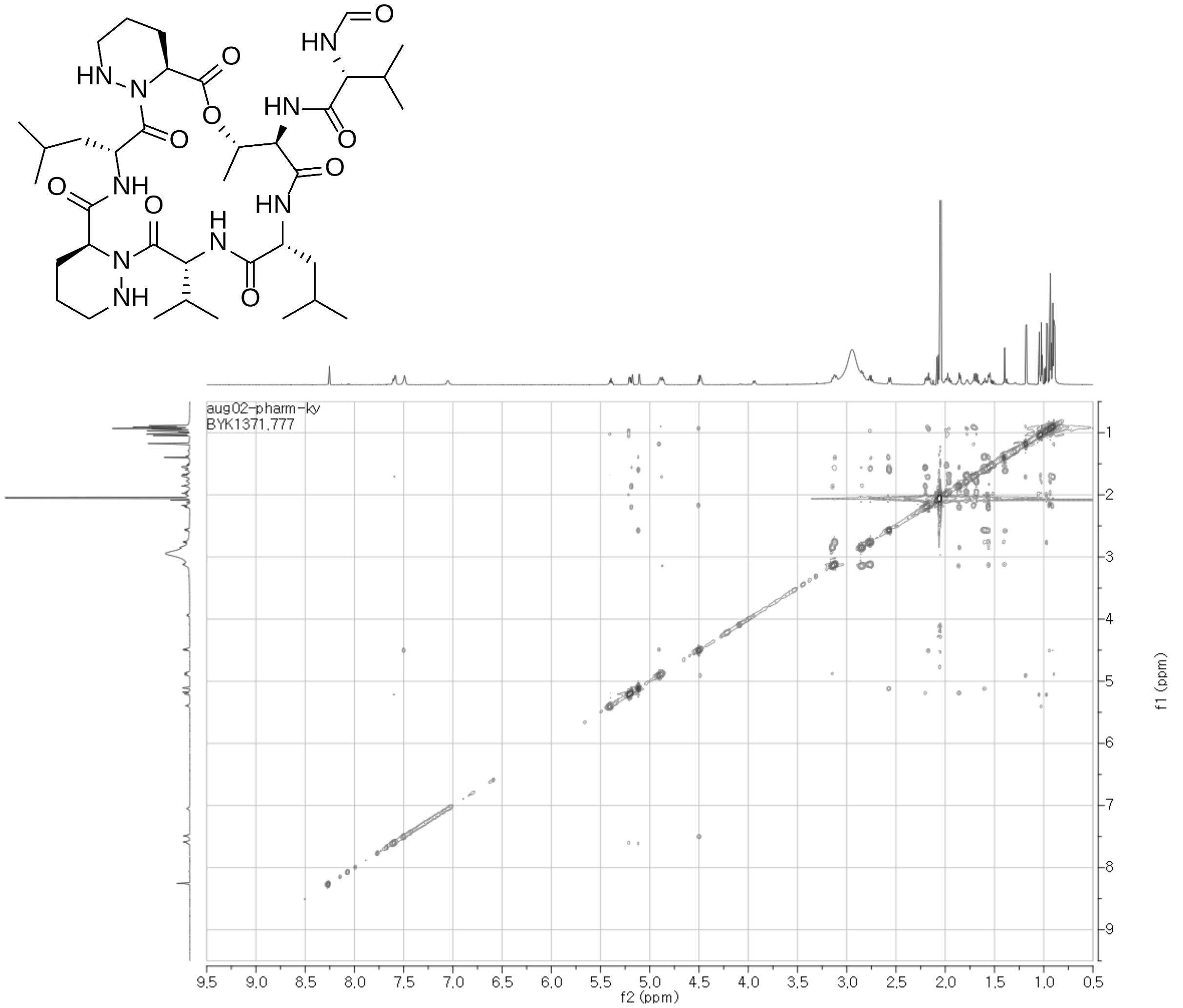

Figure S18. HSQC spectrum (850 MHz) of **2** in acetone–d_6.

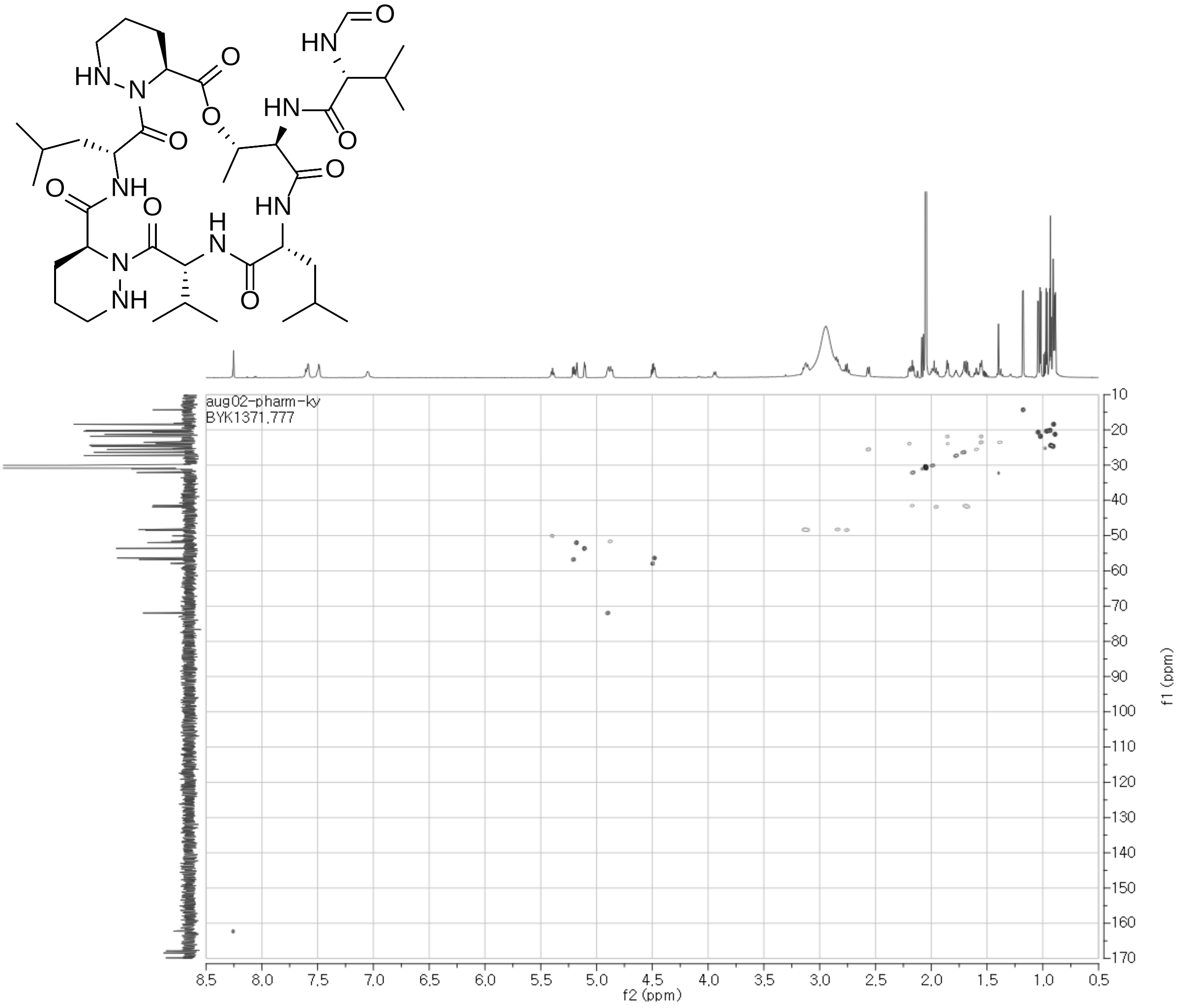

Figure S19. HMBC spectrum (850 MHz) of **2** in acetone–d_6.

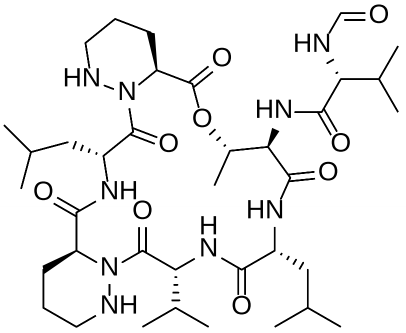

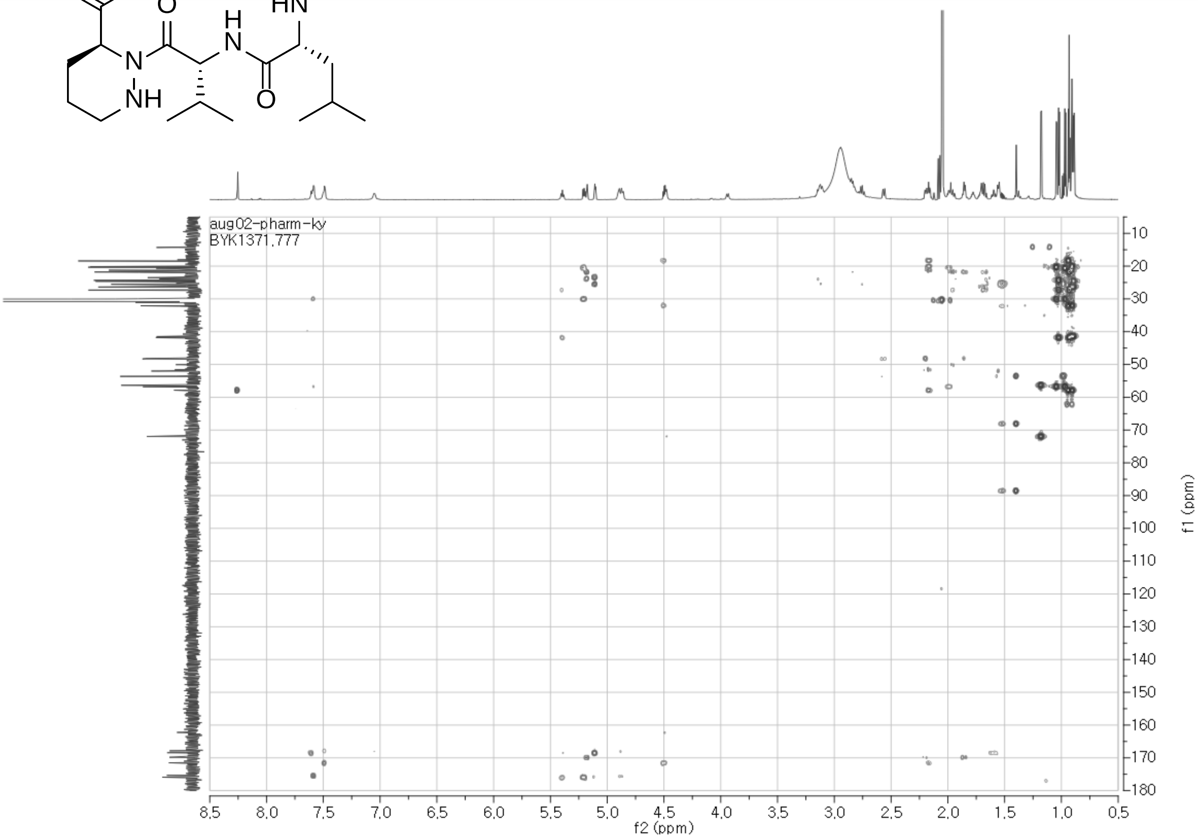

Figure S20. ROESY spectrum (850 MHz) of **2** in acetone–d_6.

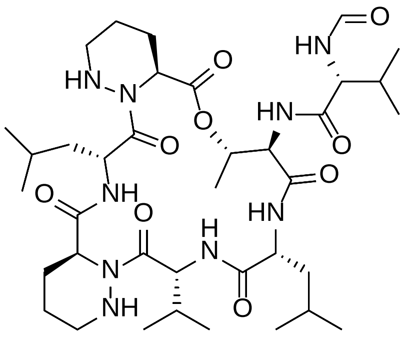

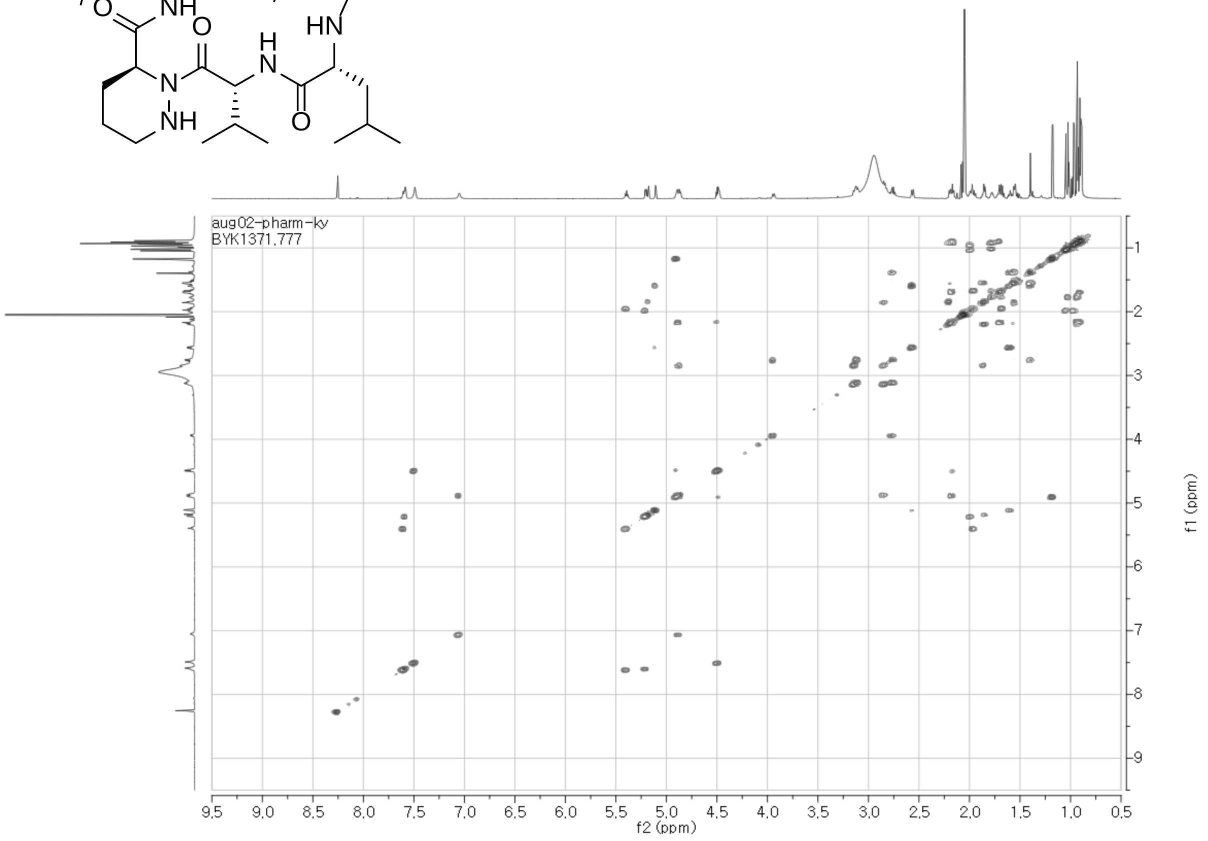

Figure S21. TOCSY spectrum (850 MHz) of **2** in acetone–d_6.

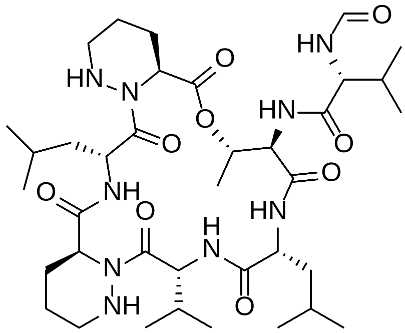

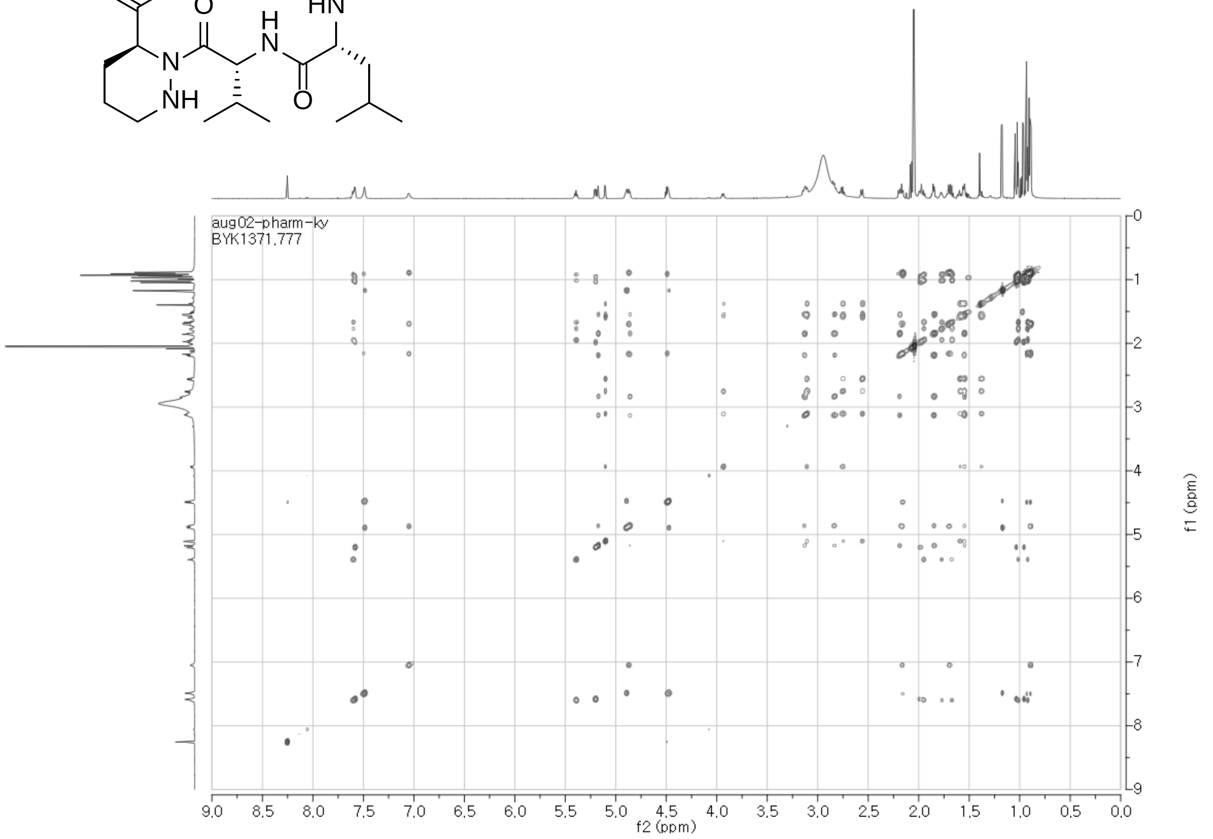

Figure S22. ^{1}H NMR spectrum (600 MHz) of **3** in acetone–d_6.

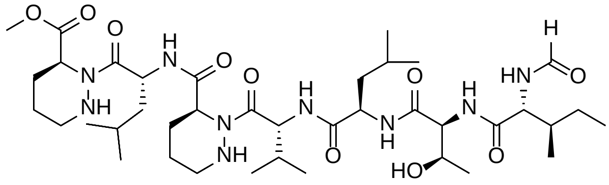

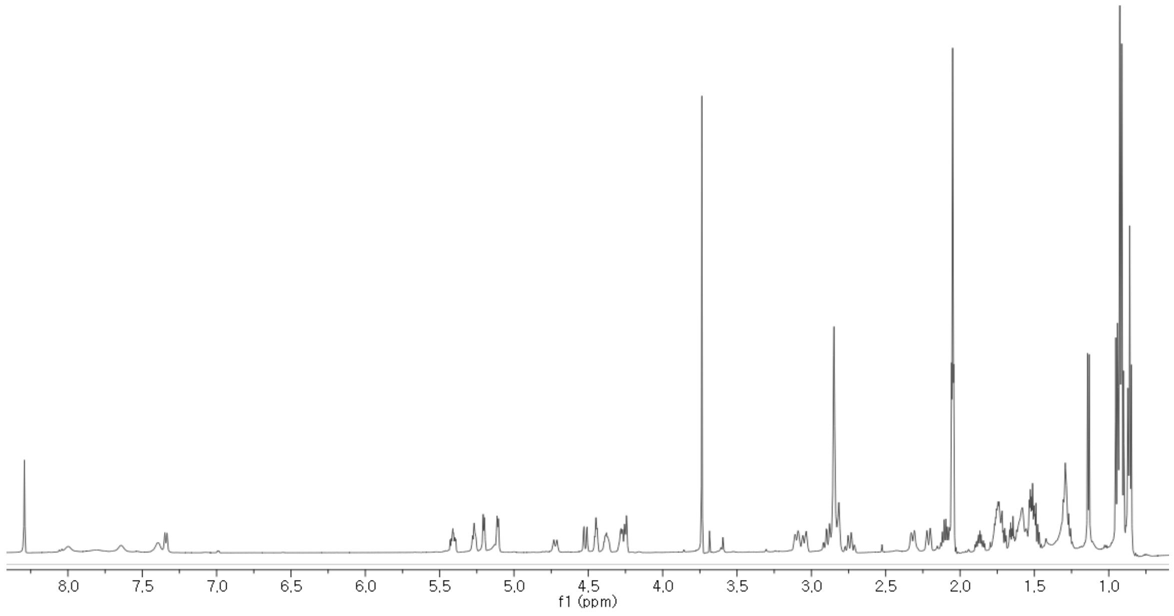

Figure S23. ^{13}C NMR spectrum (150 MHz) of **3** in acetone–d_6.

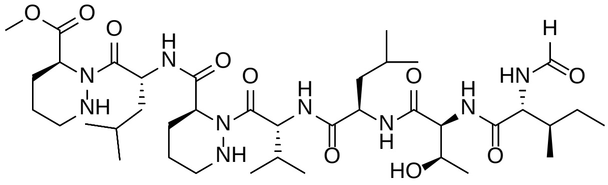

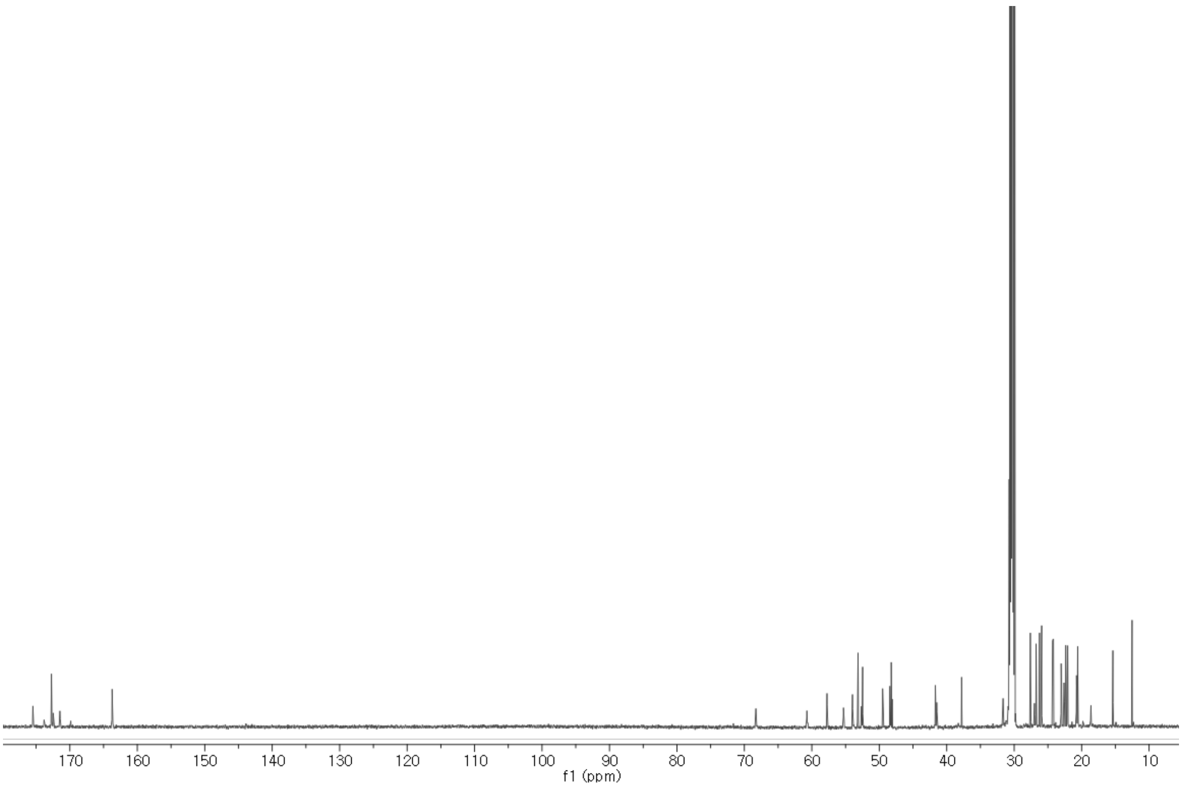

Figure S24. COSY spectrum (600 MHz) of **3** in acetone–d_6.

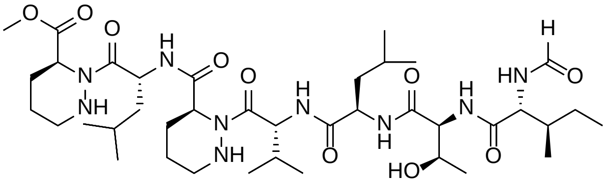

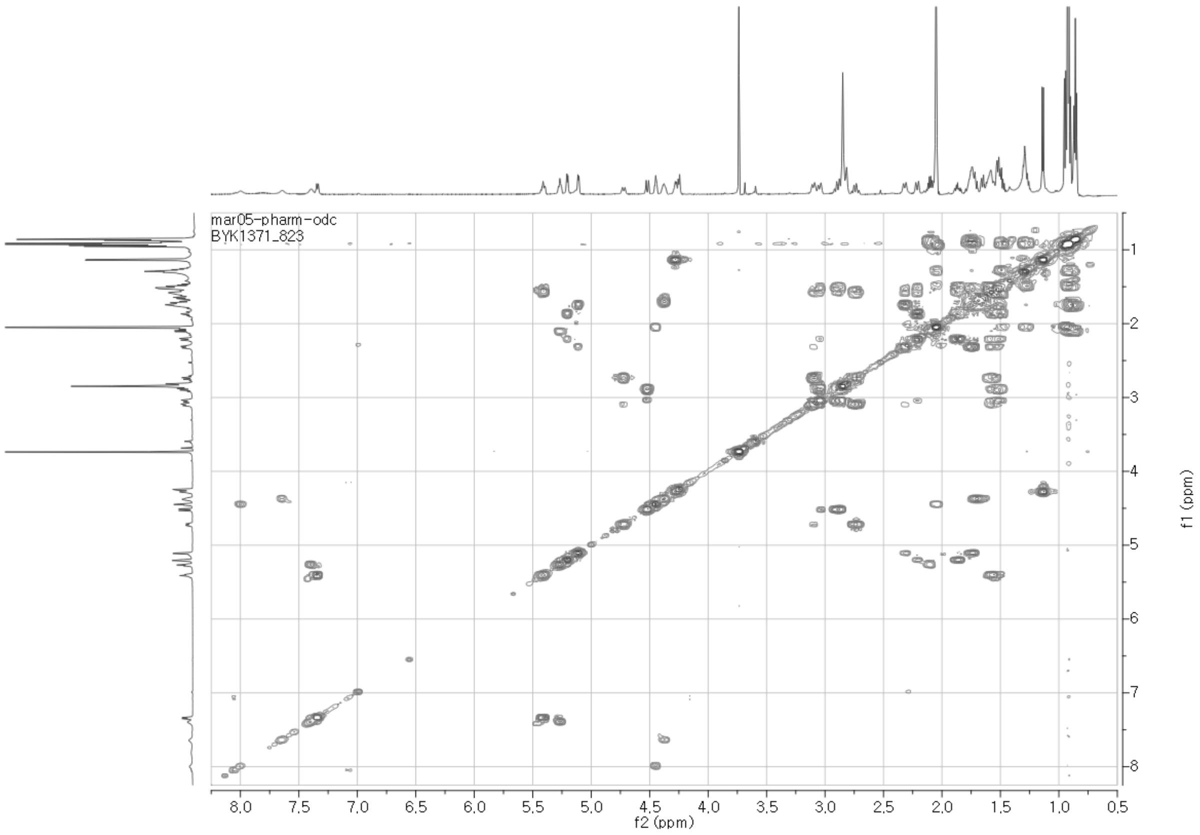

Figure S25. HSQC spectrum (600 MHz) of **3** in acetone–d_6.

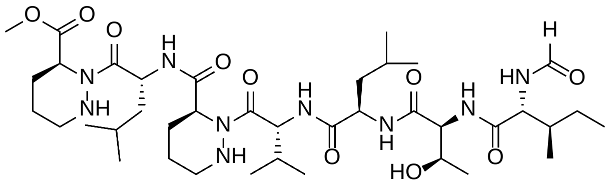

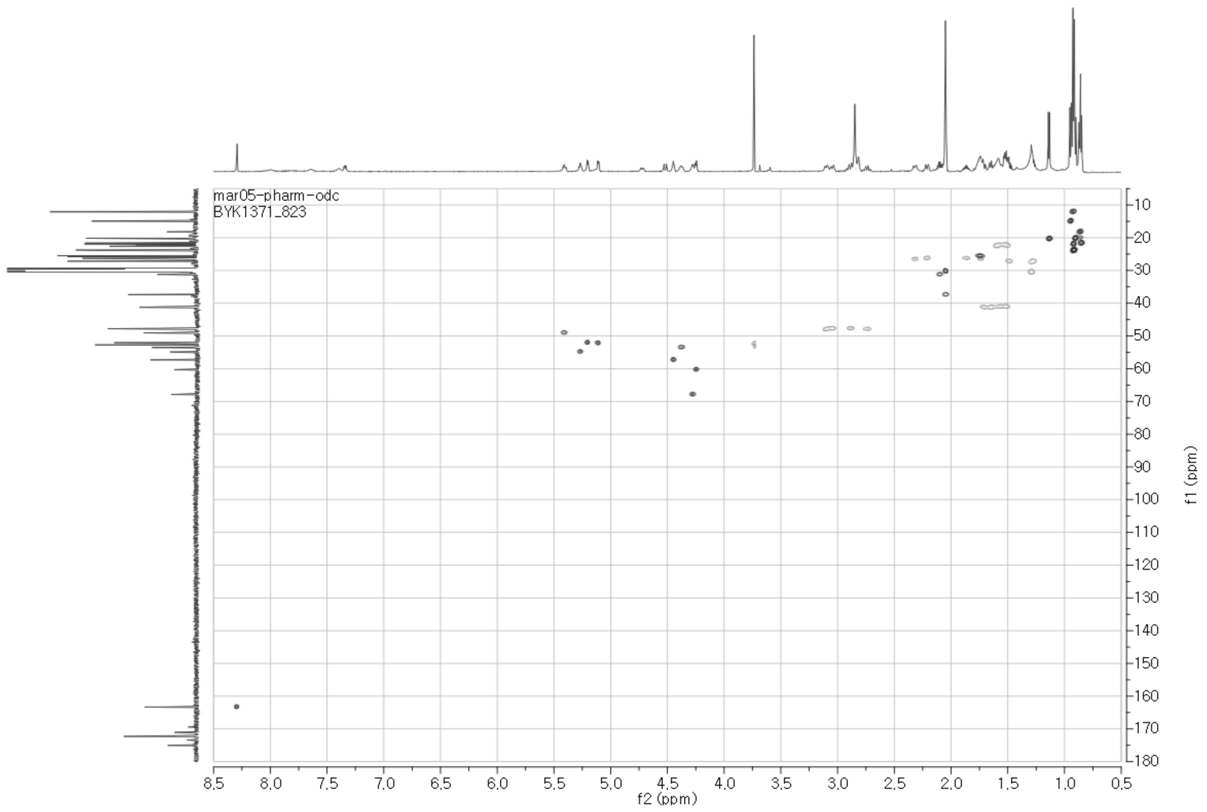

Figure S26. HMBC spectrum (600 MHz) of **3** in acetone–d_6.

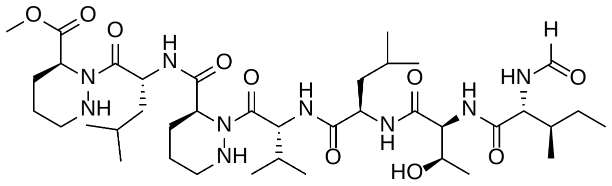

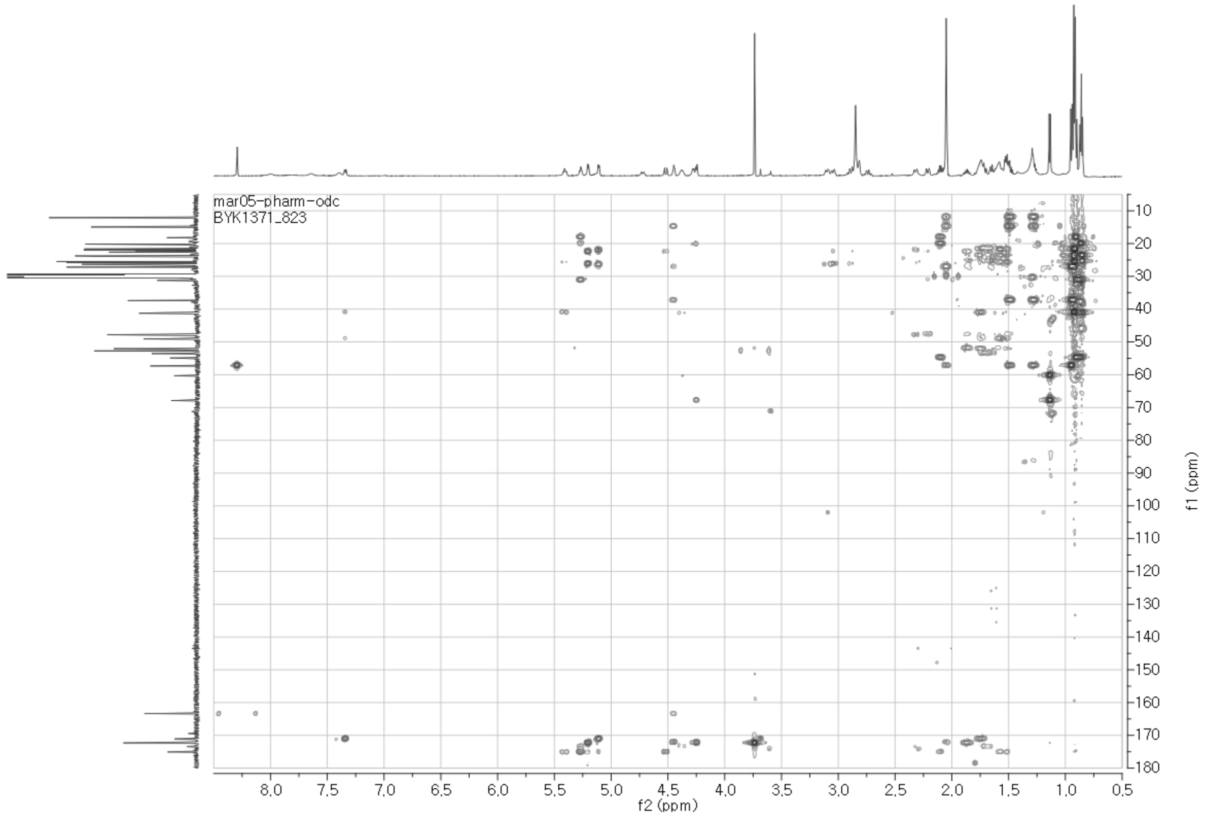

Figure S27. TOCSY spectrum (600 MHz) of **3** in acetone–d_6.

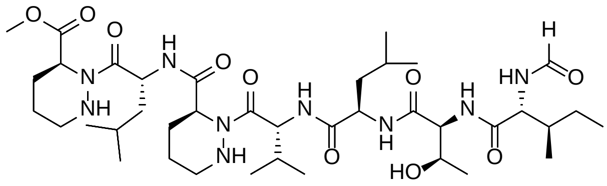

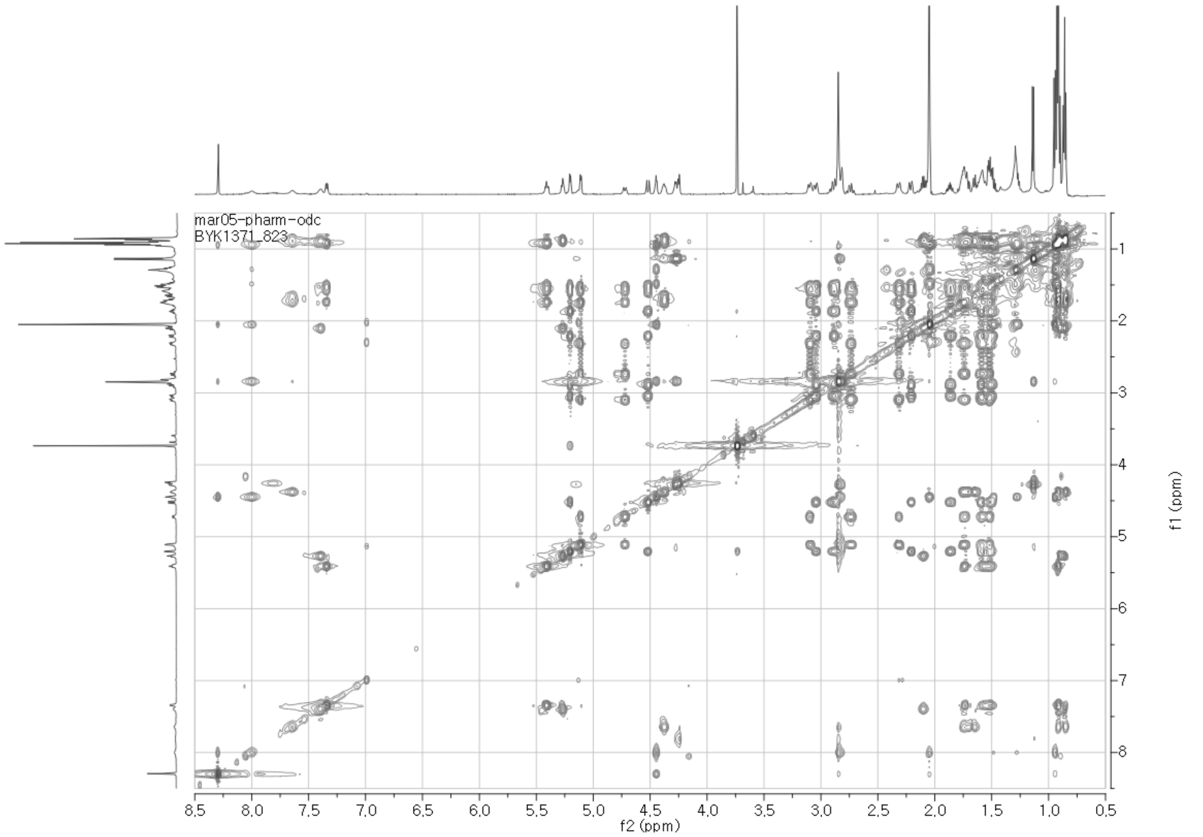

Figure S28. MS/MS data of **3**.

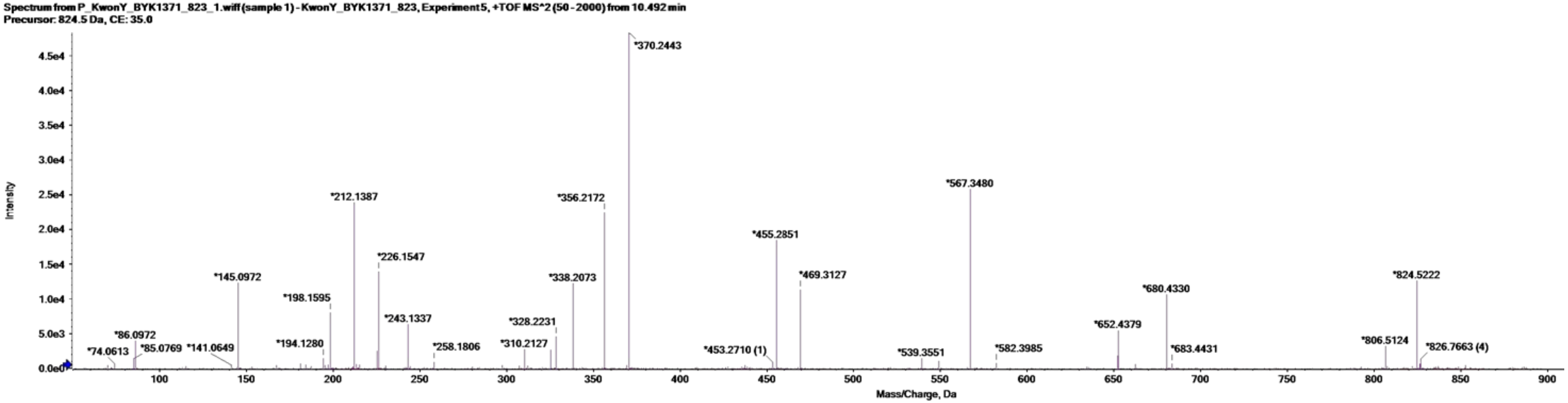

Figure S29. LC/MS chromatogram of (a) L- and (b) D-FDAA derivatives of **1**.

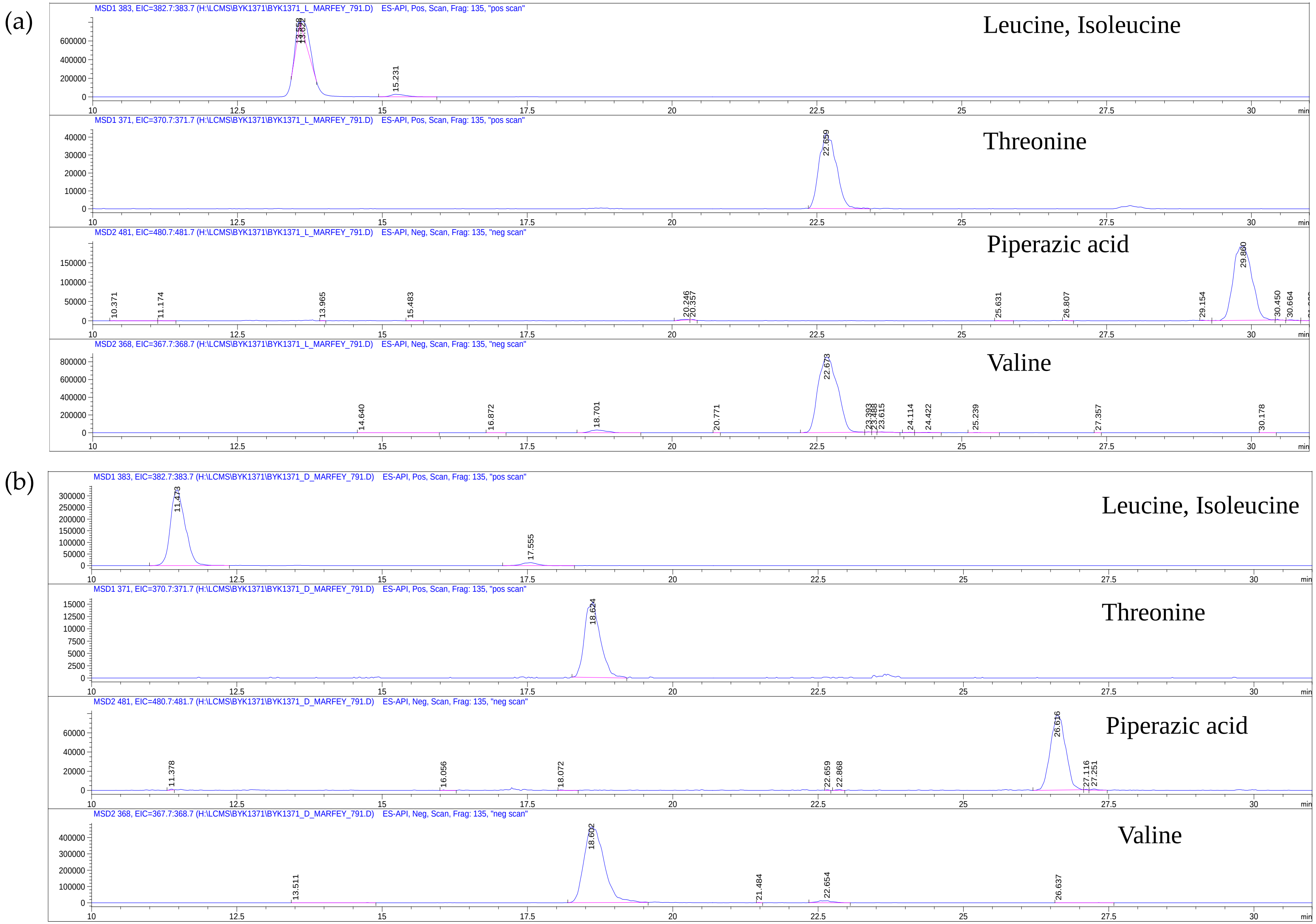

Figure S30. LC/MS chromatogram of (a) L- and (b) D-FDAA derivatives of **2**.

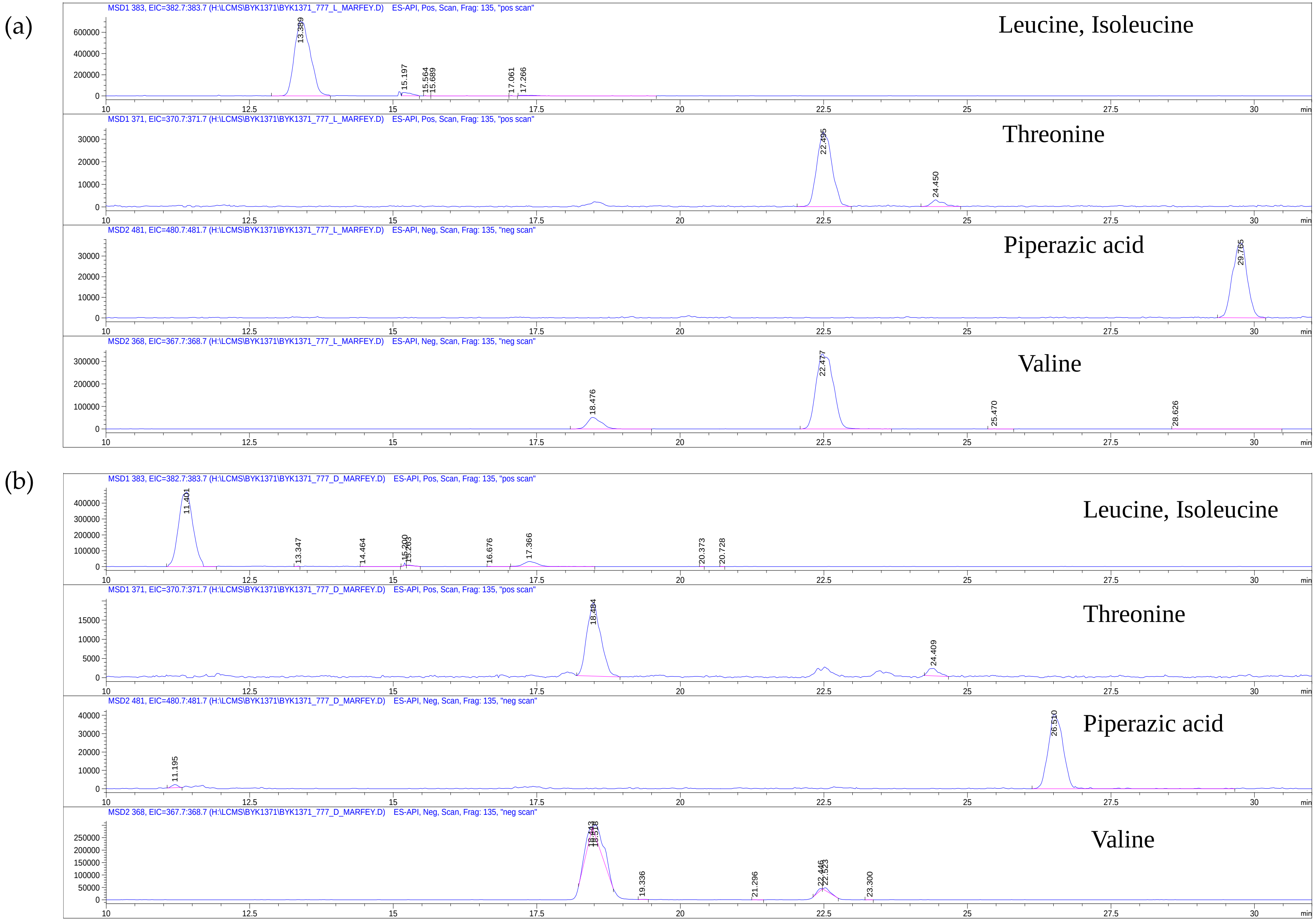

Figure S31. LC/MS chromatogram of GITC derivatives of (a) **1**, (b) authentic D-Ile, (c) D-*allo*-Ile, (d) coinjection of GITC derivatives of **1** with authentic (d) D-Ile and (e) D-*allo*-Ile.

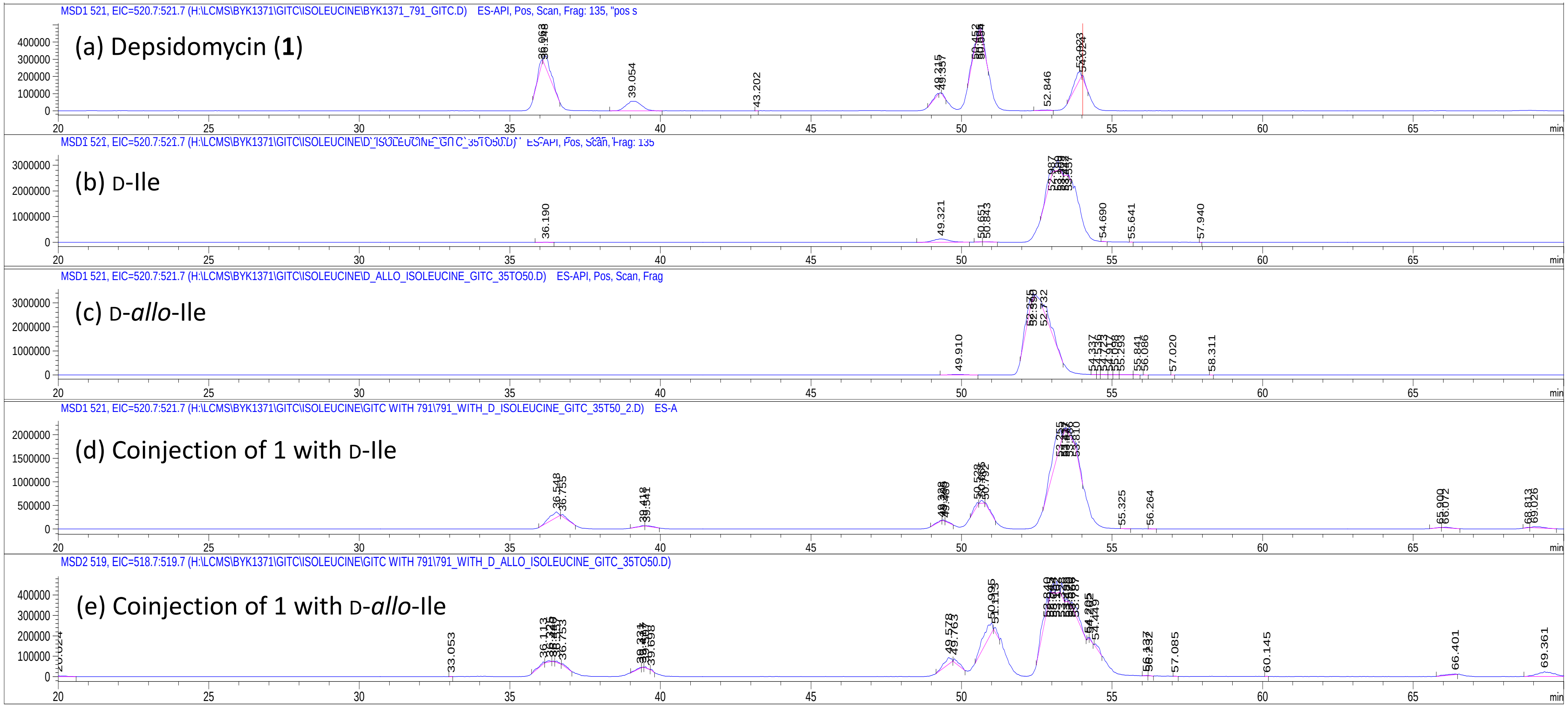

Figure S32. LC/MS chromatogram of GITC derivatives of (a) **1**, (b) authentic D-Thr, (c) L-Thr, (d) D-*allo*- and L-*allo*-Thr, and coinjection of GITC derivatives of authentic D-Thr with those of (e) **1** and (f) **2**.

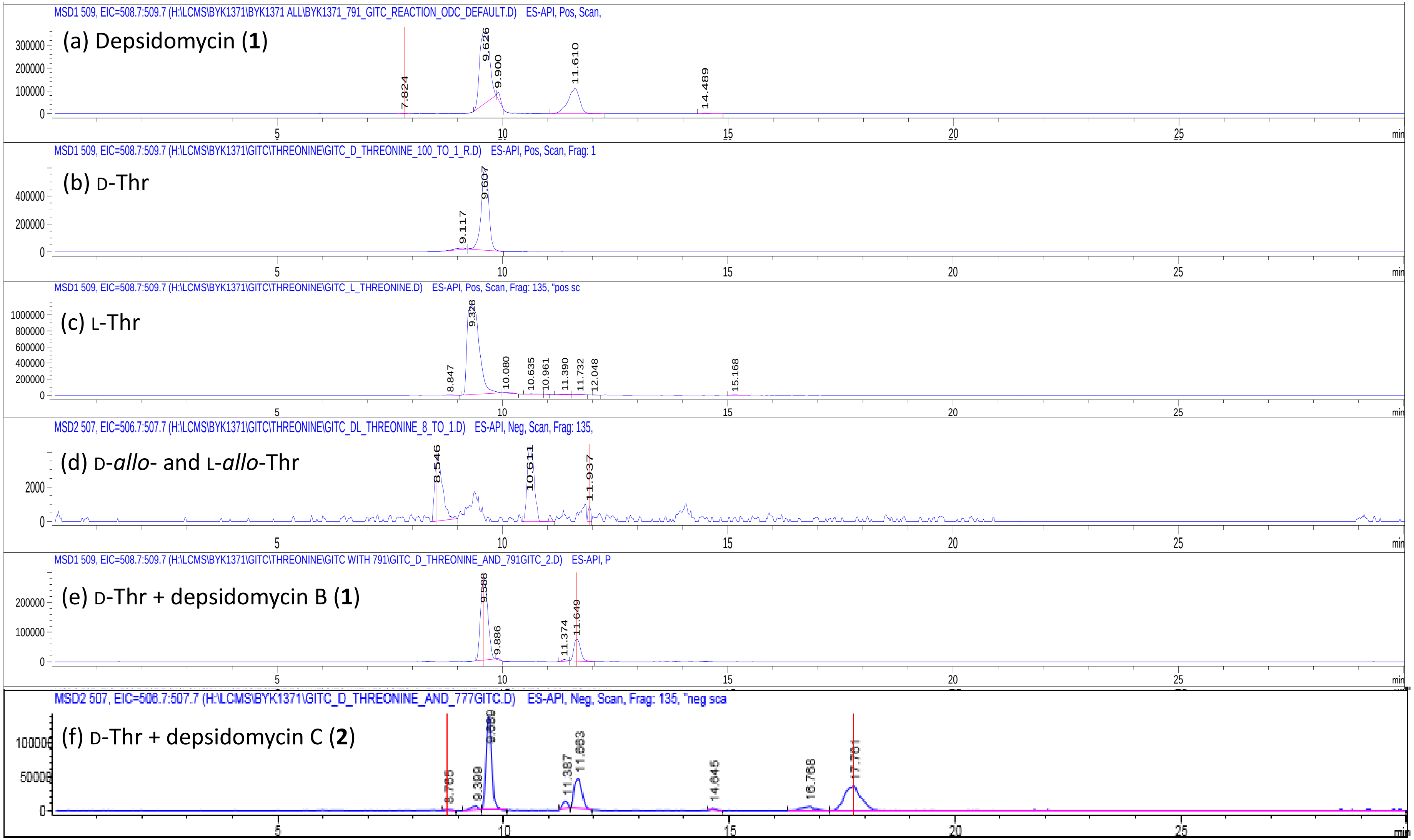

Figure S33. Positive control data of wound healing assay using sunitinib.

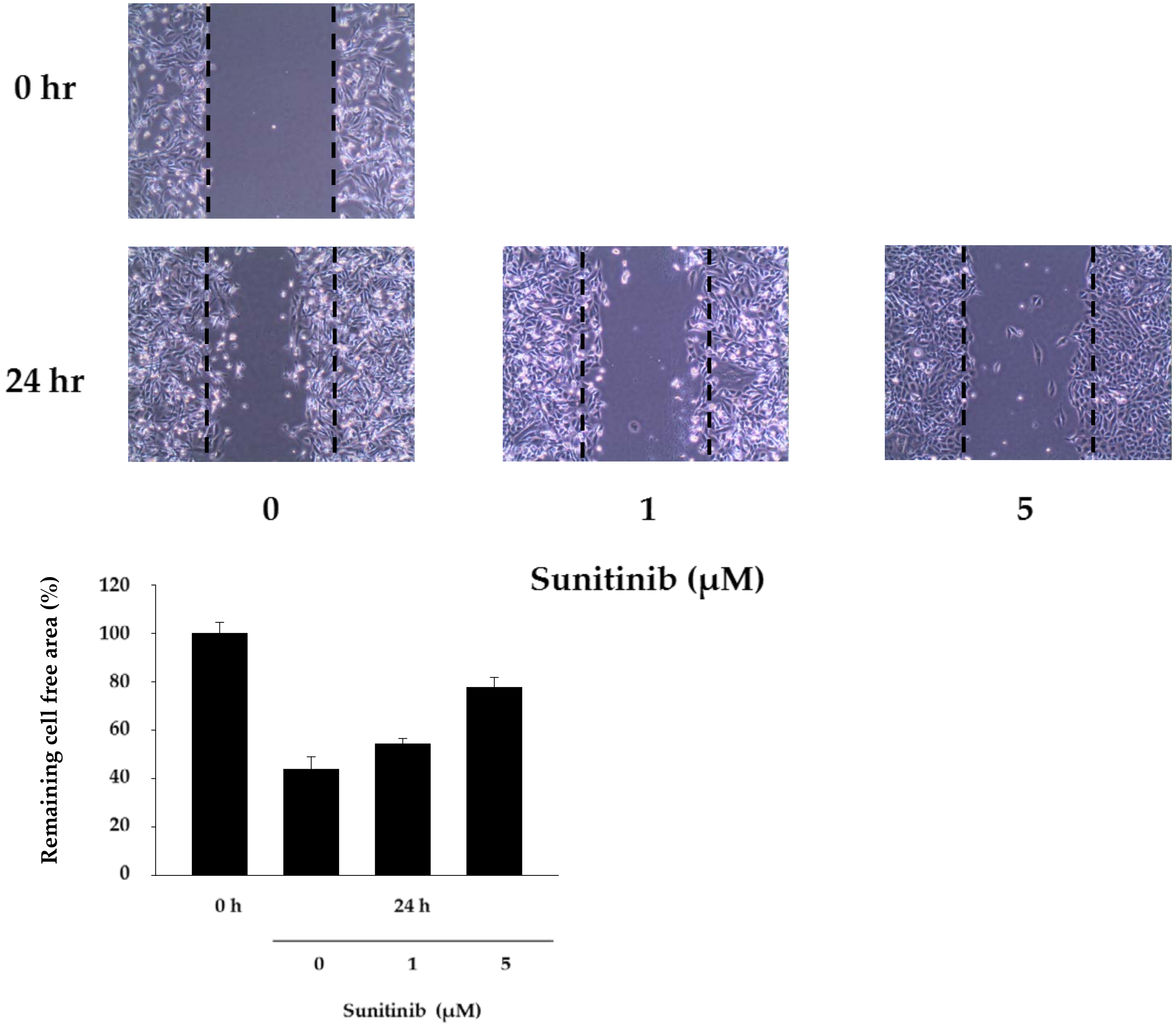

Figure S34. Time-course LC/MS analysis of the culture of the strain BYK1371. Depsidomycins B and C (**1**-**2**) were detected at 15.4 and 14.6 min, respectively.

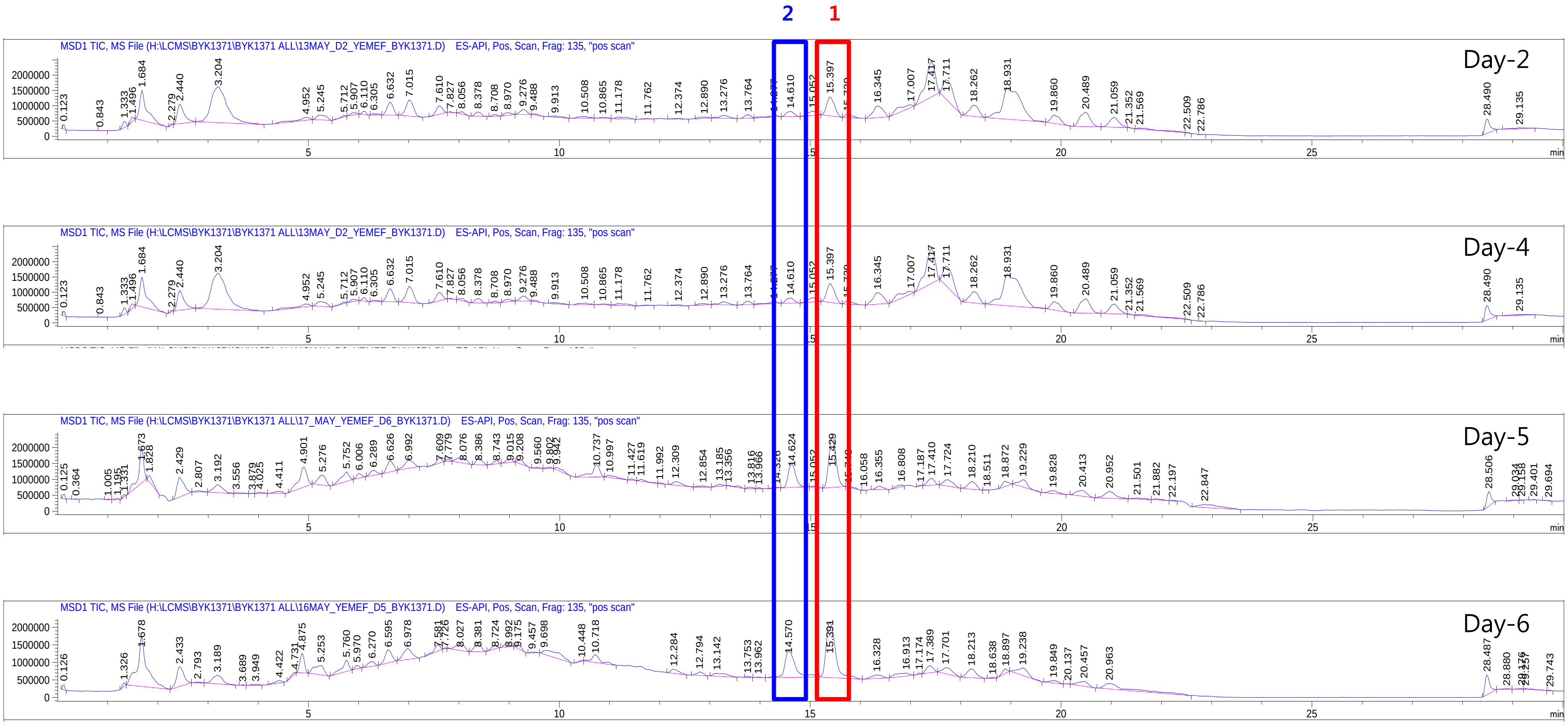

Figure S35. Phylogenetic relationships of single strain BYK 1371 and related S*treptomyces* taxa.

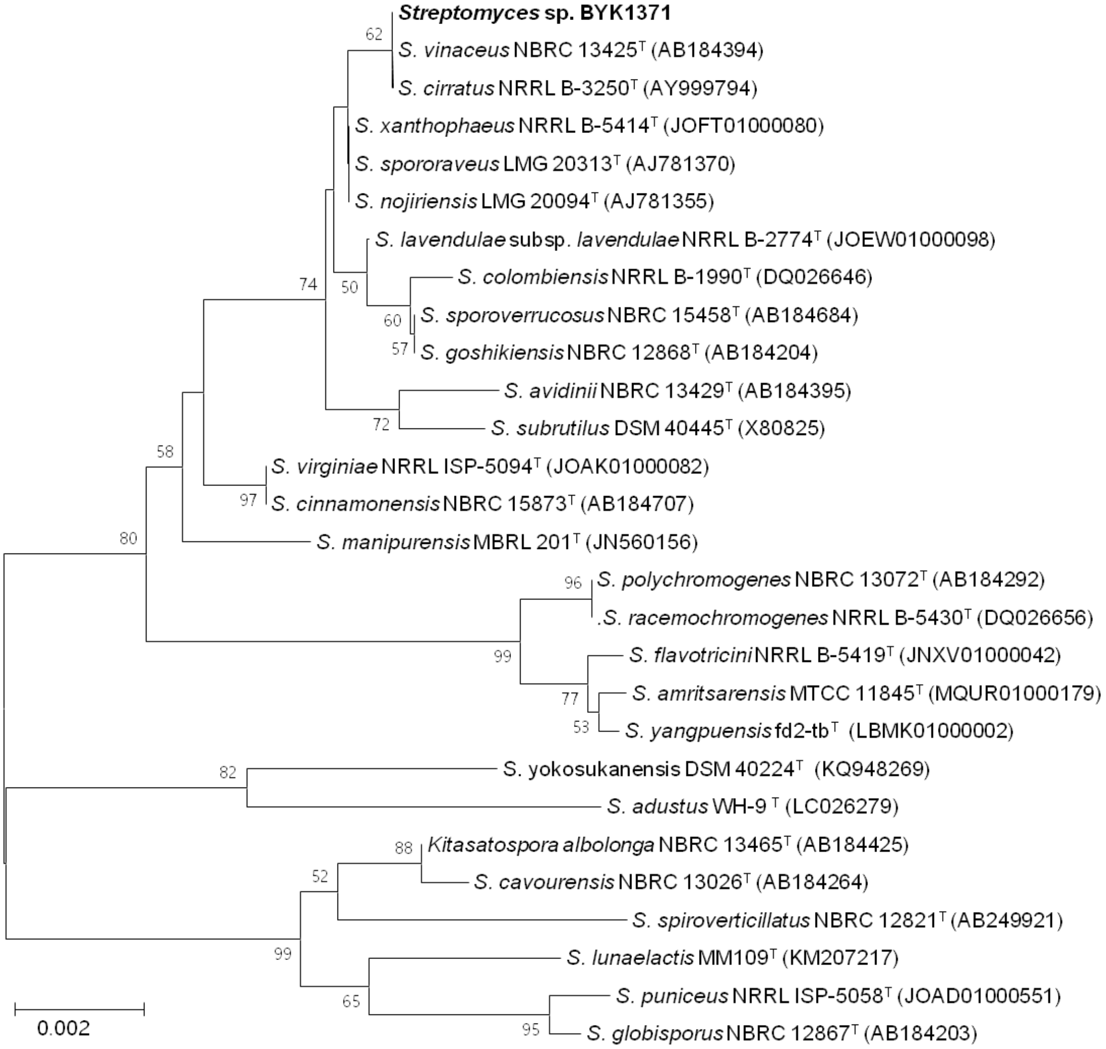

The evolutionary history was inferred using the Neighbor-Joining method [1]. The tree based on partial 16S rRNA gene sequences (about 1,400 nucleotides) showing the relationships between isolate BYK1371 and the closely related type strains of the genus *Streptomyces*. Numbers at the nodes indicate levels of bootstrap support (%) above 50% based on a neighbour-joining analysis of 1,000 resampled datasets [2]. Genbank accession numbers are indicated in parentheses. The scale bar indicates the number of substitutions per nucleotide position. The evolutionary distances were computed using the Kimura 2-parameter method [3] and are in the units of the number of base substitutions per site. The analysis involved 33 nucleotide sequences. All positions containing gaps and missing data were eliminated. Evolutionary analyses were conducted in MEGA X [4].

Table S1. ^{1}H and ^{13}C NMR data for **1** in pyridine-d_5.

**positon**	**Depsidomycin B (1)**	
	**δ_C, type** [a]	**δ_H, mult (*J* in Hz)** [a]
1	169.5, C	
2	51.5, CH	5.43, br d (3.5)
3a	21.6, CH_2	1.30, m
3b		1.88, m
4a	21.3, CH_2	1.60, m
4b		2.11, m
5a	48.2, CH_2	2.66, m
5b		3.06, m
5-NH		5.69, br d (12.0)
6	175.5, C	
7	49.9, CH	6.02, dd (10.5, 10.0)
7-NH		8.39, br d (10.0)
8a	41.7, CH_2	2.10, m
8b		2.54, br dd (10.0, 10.0)
9	27.0, CH	2.15, m
10	24.1, CH_3	1.12, d (6.5)
11	21.5, CH_3	1.24, d (6.5)
12	168.6, C	
13	53.1, CH	5.77, br d (5.0)
14a	25.4, CH_2	1.65, m
14b		2.91, br d (11.0)
15a	23.3, CH_2	1.48, d (9.0)
15b		1.63, m
16a	48.1, CH_2	3.12, m
16b		2.78, m
16-NH		4.44, br d (13.0)
17	176.3, C	
18	56.8, CH	5.58, m
18-NH		9.33, br d (5.0)
19	29.2, CH	2.33, m
20	20.2, CH_3	1.05, m
21	20.5, CH_3	1.07, m
22	175.3, C	
23	51.5, CH	5.38, m
23-NH		7.61, br d (10.0)
24a	41.0, CH_2	2.04, m
24b		2.61, m
25	26.1, CH	1.89, m
26	24.3, CH_3	0.75, m
27	20.8, CH_3	0.79, m
28	168.2, C	
29	56.2, CH	5.33, m
29-NH		9.19, d (8.0)
30	72.3, CH	5.21, br s
31	14.5, CH_3	1.34, d (6.0)
32	172.1, C	
33	56.0, CH	5.35, m
33-NH		9.44, br s
34	38.8, CH	2.23, m
35	27.3, CH_2	1.24, d (6.5)
		1.59, m
36	12.3, CH_3	0.77, m
37	15.4, CH_3	1.00, d (7.0)
38	162.2, CH	8.77, s

[a] ^{1}H and ^{13}C NMR were recorded at 800 and 212.5 MHz, respectively.

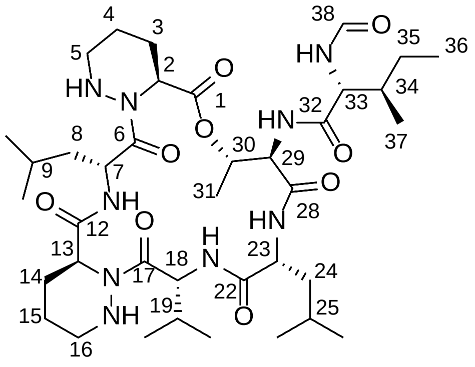

Table S2. ^{1}H and ^{13}C NMR data for **3** in Acetone-d_6.

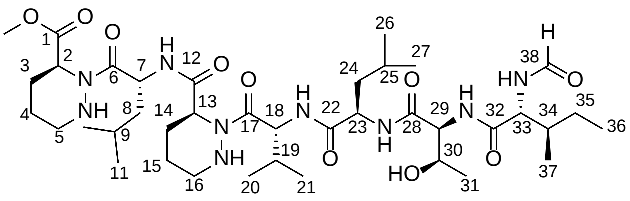

positon	3	
	δ_C, type [a]	δ_H, mult (*J* in Hz) [a]
1	172.1, C	
2	52.0, CH	5.20, d (6.0)
3a	26.1, CH_2	1.87, m
3b		2.21, m
4	21.3, CH_2	1.57, br s
5a	47.7, CH_2	3.06, m
5b		2.89, m
5-NH		4.52, br d (12.5)
6	175.1, C	
7	49.1, CH	5.41, m
7-NH		7.35, brd (8.0)
8	41.0, CH_2	1.55, m
9	25.6, CH	1.75, m
10	20.0, CH_3	0.86, m
11	23.8, CH_3	0.92, d (6.5)
12	171.1, C	
13	51.5, CH	5.11, br d (5.0)
14a	26.6, CH_2	1.74, m
14b		2.33, br d (13.5)
15a	21.4, CH_2	1.55, m
15b		1.59, br
16a	48.4, CH_2	3.10, m
16b		2.71, m
16-NH		4.72, br d (13.5)
17	175.2, C	
18	52.2, CH	5.27, m
18-NH		7.40, br s
19	31.2, CH	2.08, m
20	18.3, CH_3	0.87, d (6.5)
21	22.0, CH_3	0.92, d (6.5)
22	173.4, C	
23	53.6, CH	4.38, m
23-NH		7.64, br s
24	41.2, CH_2	1.64, m
25	26.3, CH	1.73, m
26	21.6, CH_3	0.85, m
27	20.0, CH_3	0.90, m
28	171.5, C	
29	57.2, CH	4.45, m
29-NH		7.40, d (6.0)
30	67.9, CH	4.28, m
31	20.3, CH_3	1.14, d (6.5)
32	172.2, C	
33	57.2, CH	4.45, m
33-NH		8.01, br s
34	37.3, CH	2.05, m
35	27.3, CH_2	1.28, d (6.5)
		1.49, d (6.5)
36	12.1, CH_3	0.93, m
37	15.0, CH_3	0.95, d (7.0)
38	163.3, CH	8.29, s
39	52.7, O-CH_3	3.74, s

[a] ^{1}H and ^{13}C NMR were recorded at 600 and 150 MHz, respectively.

Table S3. ^1H and ^{13}C NMR data for depsidomycin and B (**1**) in Acetone-d_6.

Positon	Depsidomycin		Depsidomycin B (1)	
	δC, type	δH, mult (*J* in Hz) [a]	δC, type	δH, mult (*J* in Hz) [b]
1	169.3, C		169.3, C	
2	51.6, CH	5.16, dd (5.0, 2.0)	51.4, CH	5.18, br s
3a	23.4, CH_2	1.86, m	23.4, CH_2	1.85, m
3b		2.19, m		2.19, m
4a	21.4, CH_2	1.55, m	21.3, CH_2	1.55, m
4b		1.86, m		1.86, m
5a	47.8, CH_2	3.14, m	47.9, CH_2	3.12, m
5b		2.85, m		2.85, m
5-NH		4.88, dd (12.0, 2.5)		4.88, m
6	175.4, C	–	175.4, C	
7	49.6, CH	5.40, m	49.7, CH	5.40, dd (10.5, 10.5)
7-NH		7.65, d (10.0)		7.62, d (10.5)
8a	41.4, CH_2	1.69, m	41.4, CH_2	1.68, m
8b		1.95, m		1.95, m
9	26.8, CH	1.78, m	26.6, CH	1.78, m
10	21.3, CH_3	1.02, d (6.5)	21.0, CH_3	1.02, d (6.5)
11	23.8, CH_3	0.93, d (6.5)	23.7, CH_3	0.93, d (6.5)
12	167.9, C	–	167.7, C	
13	53.1, CH	5.11, dd (5.5, 1.5)	52.8, CH	5.12, d (4.5)
14a	25.1, CH_2	1.55, m	24.9, CH_2	1.58, m
14b		2.57, m		2.57, br d (13.5)
15a	23.0, CH_2	1.40, m	23.0, CH_2	1.39, m
15b		1.55, m		1.55, br d (10.5)
16a	48.0, CH_2	2.77, m	47.8, CH_2	2.75, m
16b		3.12, m		3.12, m
16-NH		3.94, dd (13.0, 2.0)		3.95, br d (12.5)
17	175.3, C	–	175.9, C	
18	56.3, CH	5.20, dd (10.5, 6.0)	56.2, CH	5.20, dd (10.0, 6.0)
18-NH		7.56, d (6.0)		7.59, d (6.0)
19	29.4, CH	1.98, m	29,5, CH	1.99, m
20	20.1, CH_3	1.05, d (6.5)	21.0, CH_3	1.02, d (6.5)
21	19.9, CH_3	0.97, d (6.5)	19.7, CH_3	0.97, d (6.5)
22	174.8, C	–	174.7, C	
23	51.2, CH	4.97, m	51.0, CH	4.88, m
23-NH		7.10, d (10.0)		7.07, br s
24a	41.1, CH_2	1.68, m	40.8, CH	1,68, m
24b		2.17, m		2.17, m
25	25.9, CH	1.70, m	25.8, CH	1.71, m
26	20.8, CH_3	0.89, d (7.0)	20.6, CH_3	0.89, m
27	24.1, CH_3	0.91, d (7.0)	24.0, CH_3	0.91, m
28	167.4, C		167.4, C	
29	55.9, CH	4.50, dd (6.5, 3.5)	55.8, CH	4.48, br s
29-NH		7.69, d (6.5)		7.52, br s
30	71.4, CH	4.90, dq (6.5, 3.5)	71.4, CH	4.91, m
31	13.9, CH	1.17, d (6.5)	13.6, CH_3	1.17, d (6.5)
32	171.1, C		171.7, C	
33	55.5, CH	4.76, dd (9.5, 4.5)	55.4, CH	4.66, m
33-NH		7.52, d (9.5)		7.49, br s
34	38.5, CH	1.92, m	38.1, CH	1.98, m
35a	27.0, CH_2	1.19, m	26.9, CH_2	1.21, m
35b		1.42, m		1.43, m
36	12.1, CH_3	0.89, t (7.5)	11.8, CH_3	0.90, m
37	14.7, CH_3	0.87, d (7.0)	14.6, CH_3	0.87, d (7.0)
38	161.8, CH	8.27, br s	161.8, CH	8.27, s

[a] ^1H and ^{13}C NMR were cited from the origin paper.

[b] ^1H and ^{13}C NMR were recorded at 850 and 212.5 MHz, respectively

Table S4. LC/MS analysis of the FDAA derivatives of **1** and **2**.

Depsidomycin B (**1**)				Depsidomycin C (**2**)			
Amino acid	Elution order	t_{RL} (min)	t_{RD} (min)	Amino acid	Elution order	t_{RL} (min)	t_{RD} (min)
Val (D)	$D \rightarrow L$	22.6	18.6	Val (D)	$D \rightarrow L$	22.4	18.4
Leu (D)	$D \rightarrow L$	13.6	11.4	Leu (D)	$D \rightarrow L$	13.3	11.4
Ile (D)	$D \rightarrow L$	13.6	11.4	Ile (D)	$D \rightarrow L$	13.3	11.4
Thr (D)	$D \rightarrow L$	22.6	18.6	Thr (D)	$D \rightarrow L$	22.4	18.4
Pip (*S*)	$D \rightarrow L$	29.8	26.6	Pip (*S*)	$D \rightarrow L$	29.7	26.5

References.

1. Saitou N.; Nei M. The neighbor-joining method: A new method for reconstructing phylogenetic trees. *Mol. Biol. Evol.* **1987**, *4*, 406-425.
2. Felsenstein J. Confidence limits on phylogenies: An approach using the bootstrap. *Evolution* **1985**, *39*, 783-791.
3. Kimura M. A simple method for estimating evolutionary rate of base substitutions through comparative studies of nucleotide sequences. *J. Mol. Evol.* **1980**, *16*, 111-120.
4. Kumar S.; Stecher G.; Li M., Knyaz C.; Tamura K. MEGA X: Molecular evolutionary genetics analysis across computing platforms. *Mol. Biol. Evol.* **2018** (https://doi.org/10.1093/molbev/msy096).
